# Long non-coding RNA MFSD4A-AS1 promotes lymphangiogenesis and lymphatic metastasis of papillary thyroid cancer

**DOI:** 10.1530/ERC-22-0221

**Published:** 2023-02-08

**Authors:** Xiaoli Liu, Chunhai Zhang, Xiaomiao Wang, Can Cui, Hanwen Cui, Baishu Zhu, Anqi Chen, Lu Zhang, Jingwei Xin, Qingfeng Fu, Gianlorenzo Dionigi, Hui Sun

**Affiliations:** 1 Division of Thyroid Surgery, China-Japan Union Hospital of Jilin University, Jilin Provincial Key Laboratory of Surgical Translational Medicine, Changchun, Jilin, China; 2 Department of Pathophysiology and Transplantation, University of Milan, Department of Surgery, IRCCS Istituto Auxologico Italiano, Milan, Italy; 3 Division of General Surgery, Endocrine Surgery Section, Istituto Auxologico Italiano IRCCS (Istituti di Ricovero e Cura a Carattere Scientifico), Milan, Italy

**Keywords:** MFSD4A-AS1, TGF-β signaling, lymph node metastasis, papillary thyroid cancer

## Abstract

Lymphatic metastasis is the leading cause responsible for recurrence and progression in papillary thyroid cancer (PTC), where dysregulation of long non-coding RNAs (lncRNAs) has been extensively demonstrated to be implicated. However, the specific lymphatic node metastatsis-related lncRNAs remain not identified in PTC yet. Lymphatic node metastatsis-related lncRNA, MFSD4A-AS1, was explored in the PTC dataset from The Cancer Genome Atlas and our clinical samples. The roles of MFSD4A-AS1 in lymphatic metastasis were investigated *in vitro* and *in vivo*. Bioinformatic analysis, luciferase assay and RNA immunoprecipitation assay were performed to identify the potential targets and the underlying pathway of MFSD4A-AS1 in lymphatic metastasis of PTC. MFSD4A-AS1 was specifically upregulated in PTC tissues with lymphatic metastasis. Upregulating MFSD4A-AS1 promoted mesh formation and migration of human umbilical vein endothelial cells and invasion and migration of PTC cells. Importantly and consistently, MFSD4A-AS1 promoted lymphatic metastasis of PTC cells *in vivo* by inducing the lymphangiogenic formation and enhancing the invasive capability of PTC cells. Mechanistic dissection further revealed that MFSD4A-AS1 functioned as competing endogenous RNA to sequester miR-30c-2-3p, miR-145-3p and miR-139-5p to disrupt the miRNA-mediated inhibition of vascular endothelial growth factors A and C, and further activated transforming growth factor (TGF)-β signaling by sponging miR-30c-2-3p that targeted TGFBR2 and USP15, both of which synergistically promoted lymphangiogenesis and lymphatic metastasis of PTC. Our results unravel novel dual mechanisms by which MFSD4A-AS1 promotes lymphatic metastasis of PTC, which will facilitate the development of anti-lymphatic metastatic therapeutic strategy in PTC.

## Introduction

Thyroid cancer is one of the most commonly seen endocrine malignancies with a clear increased incidence worldwide in recent years (Albores-Saavedra *et al.* 2007, Blomberg *et al.* 2012). Thyroid cancer can be divided into papillary thyroid cancer (PTC), follicular thyroid cancer, anaplastic thyroid cancer (ATC) and medullary thyroid cancer based on histological classification, where PTC as the most common histological type accounts for approximately 90% of all thyroid cancer types (Mao & Xing 2016). Although the vast majority of PTC patients have an excellent prognosis with 5-year survival rate exceeding 95% after treated by surgical removal, followed by adjuvant radioactive iodine therapy (Hay *et al.* 2002), lymph node metastasis of PTC is the principal clinical issue affecting the prognosis of PTC patients (Zhang *et al.* 2019). Therefore, the identification of lymph node metastasis-relevant factors will facilitate early detection of lymph node metastasis and the development of anti-lymph node metastasis therapeutic strategy in PTC patients.

Lymph node metastasis is a dynamic and sequential biological process arising from primary tumors (Stacker *et al.* 2014). Once tumor cells metastasize to and colonized in the lymph node, tumor-secreted cytokines, such as vascular endothelial growth factors C (VEGFC) and VEGFD, induce proliferation and growth of new lymphatic vessels (lymphangiogenesis) by binding to VEGF receptors on lymphatic endothelial cells, which further promotes the tumor cells metastasizing to distant lymph nodes in the next echelon in an orderly sequence through lymphaticovenous connections to the general systemic circulation (Nathanson 2003). Lymphangiogenesis is characterized by tumor cell-induced proliferation and migration of lymphatic endothelial cells and the formation of new lymphatic capillaries in which VEGFs are reported to play the most important role (Joukov *et al.* 1996). Of note, several lines of evidence have shown that VEGFC plays a critical role in lymphangiogenesis of PTC, and overexpression of VEGFC predicted higher lymphatic metastasis risk in PTC patients (Yu *et al.* 2005, Yu *et al.* 2008). Importantly, inhibition of VEGFs signaling can suppress tumor lymphangiogenesis and metastasis to regional and distant lymph nodes in a variety of cancer types (He *et al.* 2002, Nakamura *et al.* 2004, Roberts *et al.* 2006, Burton *et al.* 2008). Hence, further investigation of the VEGFs regulator might provide a potential therapeutic strategy for PTC patients with lymph node metastasis.

The long non-coding RNAs (lncRNAs) are a class of ncRNA with lengths of more than 200 nucleotides and are implicated in multiple biological processes, including as enhancers to regulate the target genes transcription as scaffold molecules to modulate proteins/genes or proteins/proteins interactions (Ponting *et al.* 2009, Salehi *et al.* 2017). Furthermore, a great deal of attention has focused on the role of lncRNAs as competing endogenous RNAs (ceRNA) to sequester target miRNAs to disrupt the miRNAs-mediated degradation of target genes (Karreth *et al.* 2011), and dysregulation of miRNAs has been well established to promote tumor tumorigenesis, progression and metastasis in numerous cancer types (Zhang *et al.* 2018, Ren *et al.* 2017*a*, *b*, Dai *et al.* 2019, Peng *et al.* 2019). Furthermore, the pivotal role of lncRNAs in the development, progression and metastasis of various types of cancer has seized great momentum in accumulating studies (Lang *et al.* 2020, Liu *et al.* 2020, Chen *et al.* 2020*c*, Lang *et al.* 2021), including lymph node metastasis (Chen *et al.* 2018, Chen *et al.* 2020*a*). Notably, several independent studies have shown that lncRNAs may hold clinical applicable value as the potential predictive markers for early detection of lymph node metastasis in PTC (Feng *et al.* 2019, Zhuang *et al.* 2019, Li *et al.* 2020). However, the functional role of lncRNAs in lymphatic metastasis of PTC *in vivo*, as well as the underlying mechanism is still largely unknown. Therefore, an in-depth dissection of the functional role of lncRNAs and clarification of the underlying mechanism in lymphatic metastasis of PTC will deepen our understanding about the clinical and biological significance of lncRNAs in lymphatic metastasis of PTC.

In the current study, our findings combined with the analytic results of PTC dataset from The Cancer Genome Atlas (TCGA) revealed that lncRNA MFSD4A-AS1 was specifically upregulated in PTC tissues with lymph node metastasis. Gain- and loss-of-function assays showed that upregulating MFSD4A-AS1 promoted, while silencing MFSD4A-AS1 suppressed lymphatic metastasis of PTC cells *in vivo*, and proliferation and migration of human umbilical vein endothelial cells (HUVECs) and invasion and migration of PTC cells *in vitro*. Mechanistic investigations further demonstrated that upregulating MFSD4A-AS1 promoted lymphatic metastasis of PTC by sequestering miR-30c-2-3p, miR-145-3p and miR-139-5p to disrupt the miRNAs-mediated inhibition of VEGFA and VEGFC and inactivation of transforming growth factor (TGF)-β signaling. Collectively, our results provide experimental evidence regarding the clinical significance and biological role of MFSD4A-AS1 in lymphatic metastasis of PTC, which will facilitate early detection of lymph node metastasis and the development of anti-lymph node metastasis therapeutic methods in PTC patients.

## Materials and methods

### Cell lines and cell culture

Normal primary thyroid follicular epithelial (PTFE) cells were purchased from Procell (Procell Life Science & Technology Co., Ltd., Wuhan, China). Thyroid cancer cell lines, including PTC cell lines (B-CPAP, K1 and KTC-1) and ATC cell lines (BHT-101, CAL-62, KMH-2 and 8305C), were obtained from Cell Bank of Shanghai Institute of Cell Biology, Chinese Academy of Sciences (Shanghai, China). PTFE cells were cultured in CM-H023 medium (Procell), and thyroid cancer cell lines were cultured in RPMI-1640 medium (Life Technologies) supplemented with penicillin G (100 U/mL), streptomycin (100 mg/mL) and 10% fetal bovine serum (FBS, Life Technologies). All cell lines were cultured at 37°C in a humidified atmosphere with 5% CO_2_.

### Patients and tumor tissues

A total of 48 paired PTC tissues and the matched adjacent normal tissues, and 54 separate PTC tissues were obtained during surgery at the Division of Thyroid Surgery, China-Japan Union Hospital of Jilin University, between January 2016 and December 2020 (Supplementary Table 1, see section on supplementary materials given at the end of this article). Patients were diagnosed based on clinical and pathological evidence, and the specimens were immediately snap-frozen and stored in liquid nitrogen tanks. For the use of these clinical materials for research purposes, prior patients’ consents and approval from the Institutional Research Ethics Committee of Jilin University were obtained.

### Plasmid and transfection

Human MFSD4A-AS1 cDNA (Vigene Biosciences, Shandong, China) was cloned into the pcDNA3.1(+) or GV350 plasmid (GenChem, Shanghai, China). Knockdown of endogenous MFSD4A-AS1 was performed by cloning two short hairpin RNA (shRNA) oligonucleotides into the GV493 vector (GenChem). The sequences of the two separate shRNA fragments are listed in Supplementary Table 2. The 3′UTR regions of VEGFA and VEGFC and the region of MFSD4A-AS1 sequences targeted by miR-30c-2-3p, miR-145-3p or miR-139-5p were PCR-amplified from genomic DNA and cloned into pmirGLO vectors (Promega), and the list of primers used in cloning reactions was provided in Supplementary Table 2. miR-30c-2-3p, miR-145-3p and miR-139-5p agomirs was synthesized and purified by RiboBio. The (CAGAC) 12/pGL3 TGF-β/Smadresponsive luciferase reporter plasmid and the control plasmids (Clontech, Kusatsu, Shiga, Japan) were used to quantitatively assess the transcriptional activity of TGF-β signaling components. Transfection of plasmids was performed as previously described (Wu *et al.* 2018).

### RNA extraction, reverse transcription and real-time PCR

RNA from tissues and cells was extracted (TRIzol, Life Technologies) according to the manufacturer’s instructions. Messenger RNA (mRNA), lncRNA and miRNA were reverse transcribed from the total RNA using the Revert Aid First Strand cDNA Synthesis Kit (Thermo, Carlsbad, California, USA) according to the manufacturer’s protocol. Complementary DNA was amplified and quantified on the ABI 7500HT system (Applied Biosystems) using SYBR Green I (Applied Biosystems). The primers used in the reactions are listed in Supplementary Table 3. Real-time PCR was performed as described previously (Miller *et al.* 2015). Glyceraldehyde-3-phosphate dehydrogenase (GAPDH) was used as endogenous control for mRNA and lncRNA. Relative fold expressions were calculated with the comparative threshold cycle (Dai *et al.* 2017).

### Western blotting analysis

Western blot was performed according to a standard method, as described previously (Zhang *et al.* 2017*b*). Antibodies against TGFBR2 (mouse mAb, Cat#: 66636-1-Ig, 1:2000), USP15 (Mouse mAb, Cat#: 67557-1-Ig, 1:5000) and phosphorylated SMAD3 (Rabbit mAb, Cat#: ab52903, 1:5000) were purchased from Proteintech and Abcam. The membranes were stripped and reprobed with an α-tubulin (mouse mAb, Cat#: ab7291, 1:5000) and p84 (Rabbit mAb, Cat#: ab131268, 1:2000) antibody as the loading control for cytoplasm and nucleus.

### Invasion and migration assays

The invasion and migration assays were performed as described previously (Ren *et al.* 2013). The invasion assay was performed by using Transwell chamber consisting of 8‑mm membrane filter inserts (Corning) coated with Matrigel (BD Biosciences). 1.5 × 10^5^ K1 and B-CPAP cells were added to the upper chamber, and the lower chamber was filled with the medium with 10% FBS. The experiments were repeated three times, and the cell count was performed under a microscope (×100).

### Cell counting kit-8 analysis

2 × 10^3^ K1 and B-CPAP cells were seeded into 96-well plates, and the specific staining process and methods were performed according to the previous study (Zhang *et al.* 2017*a*). The experiments were repeated three times.

### Colony formation assay

The K1 and B-CPAP cells were trypsinized as single cell and suspended in the media with 10% FBS. The indicated cells (300 cells per well) were seeded into of 6-well plate for ~10–14 days. Colonies were stained with 1% crystal violet for 10 min after fixation with 10% formaldehyde for 5 min. Plating efficiency was calculated as previously described (Wang *et al.* 2016). Different colony morphologies were captured under a light microscope (Olympus). The experiments were repeated three times.

### Tube formation of human umbilical vein endothelial cells

Precooled Matrigel (Corning) was added into wells of a 24-well plate and polymerized for 30 min at 37°C. Human umbilical vein endothelial cells (HUVECs) (2 × 10^4^) suspended in 200 μL of conditional medium were added to each well and incubated with the supernatants from the indicated K1 and B-CPAP cells at 37°C in 5% CO_2_ for 6–12 h. The images of tube structure were captured under a 100× bright-field microscope, and quantification of tube formation was measured by the mesh and length of the completed tubes by Image View 3.7 (Jingtong, China). The experiments were repeated three times.

### Migration assay

4 × 10^4^ K1 and B-CPAP cells were seeded into the upper compartment of the 24-well Transwell permeable chambers (Corning), and the lower chamber of the Transwell was filled with completed media supplemented with 10% FBS as a chemoattractant. After incubation for 24 h, cells migrated to the bottom side of the chamber were fixed with stationary solution (methanol:acetic acid = 3:1), stained with crystal violet and photographed and quantified by counting in five random fields. The experiments were repeated three times.

### RNA immunoprecipitation

Cells were co-transfected with hemagglutinin (HA)–Ago2, followed by HA–Ago2 immunoprecipitation using HA antibody. Real-time PCR analysis of the IP material was used to test the association of the mRNA of VEGFA and VEGFC with the RNA-induced silencing complex. The detailed processes were performed as described previously (Li *et al.* 2017).

### Animal study

Eight-week-old BALB/c-nu mice were purchased from the Experimental Animal Center of the Guangzhou University of Chinese Medicine, and their care was in accordance with institution guidelines. The mice were randomly divided into three groups (*n* = 6 per group) and the indicated K1 cells (1 × 10^6^) were injected into the footpad of the mice, or subcutaneously. The primary tumors were allowed to form, then the mice were euthanized on the end-points, and the inguinal lymph nodes were excised and paraffin-embedded. Sections of the lymph nodes were subjected to H & E staining, and CD31, PDPN and Ki-67 staining for histological examination, and the tumor cell number was counted as previously described (Chen *et al.* 2020*b*). The ethic approval of animal study was obtained from The Tab of Animal Experimental Ethical Inspection of Jilin University.

### Luciferase assay

The K1 and B-CPAP cells (4 × 10^4^) were seeded in triplicate in 24-well plates and cultured for 24 h, and the luciferase reporter assay was performed as previously described (Ren *et al.* 2019). Cells were transfected with 250 ng (CAGAC) 12/pGL3 reporter luciferase plasmid or 5 ng pRL-TK Renilla plasmid (Promega) using Lipofectamine 3000 (Invitrogen) according to the manufacturer’s recommendation. Luciferase and Renilla signals were measured 36 h after transfection using a Dual Luciferase Reporter Assay Kit (Promega) according to the manufacturer’s protocol.

### Enzyme-linked immunosorbent assay

Cells (5 ×10^5^) were seeded into six-well plates and incubated for 24 h at 37°C in 5% CO_2_ atmosphere. Then, the conditional media (without FBS) was collected and used in order to determine the secretion of VEGFA and VEGFC, using the VEGF ELISA kit (Raybiotech, Guangzhou, Guangdong, China) in accordance with the manufacturer's protocol. Absorbance was measured on a Synergy 2 microplate system (BioTek) at 450 nm.

### Statistical analysis

All values are presented as means ± s.d.. Significant differences were determined using GraphPad 5.0 software. Student’s *t*-test was used to determine statistical differences between two groups when the groups are not matched, and a paired analysis is used to determine statistical differences between two groups when the groups are matched. One-way ANOVA was used to determine statistical differences between multiple testing. *P* < 0.05 was considered significant. All the experiments were repeated three times.

## Results

### lncRNA-MFSD4A-AS1 is specifically upregulated in PTC tissues with lymph node metastasis

**Figure 1:**
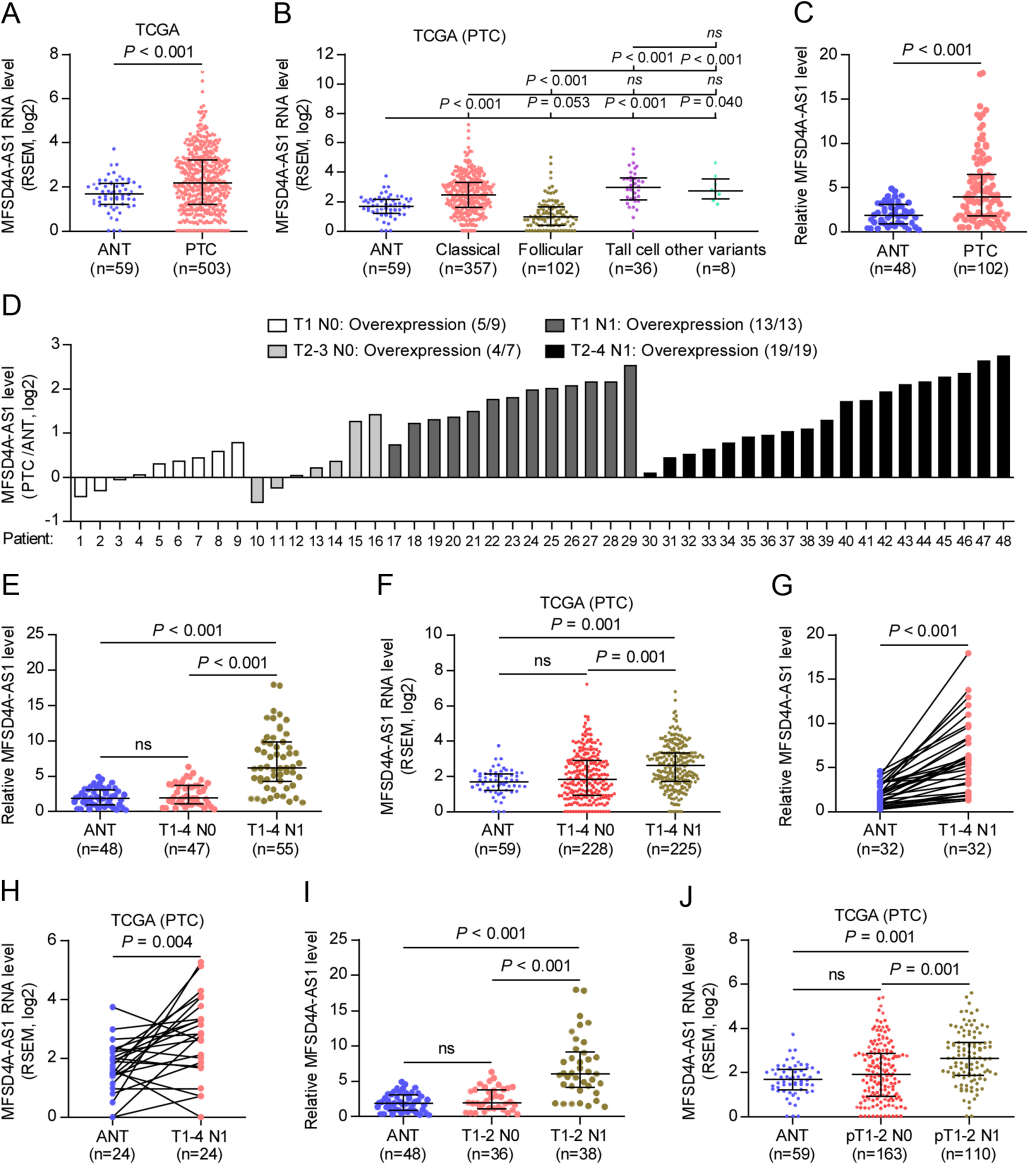
MFSD4A-AS1 is upregulated in PTC with lymph node metastasis. (A) MFSD4A-AS1 expression in 59 adjacent normal tissues (ANT) and 503 PTC tissues in the thyroid cancer dataset from TCGA. (B) MFSD4A-AS1 expression in 357 classic PTC, 102 follicular variants of PTC, 36 tall cell variants of PTC and 8 other variants, including 4 diffuse sclerosing variants, 2 cribriform morular variants, 1 columnar cell variant and 1 papillary and follicular variants, in the thyroid cancer dataset from TCGA. (C) Real-time PCR analysis of MFSD4A-AS1 expression in our 48 ANT and 102 PTC tissues without distant metastasis. GAPDH was used as endogenous control. (D) Real-time PCR analysis of MFSD4A-AS1 expression relative to their match ANT in our 48 paired PTC tissues, including 9 T1 PTC tissues without lymphatic metastasis, 7 T2–3 PTC tissues without lymphatic metastasis, 13 T1 PTC tissues with lymphatic metastasis and 19 T2–4 PTC tissues with lymphatic metastasis. GAPDH was used as endogenous control. (E) Real-time PCR analysis of MFSD4A-AS1 expression in our 48 ANT, 47 T1–4 PTC tissues without lymphatic metastasis and 65 T1–4 PTC tissues with lymphatic metastasis. GAPDH was used as endogenous control. n.s. indicates no significant difference. (F) MFSD4A-AS1 expression in 59 ANT, 230 T1–4 PTC tissues without lymphatic metastasis and 225 T1–4 PTC tissues with lymphatic metastasis in the thyroid cancer dataset from TCGA. n.s. indicates no significant difference. (G) Real-time PCR analysis of MFSD4A-AS1 expression in our 32 paired PTC tissues with lymphatic metastasis and the matched adjacent normal tissues. (H) MFSD4A-AS1 expression in 24 paired PTC tissues with lymphatic metastasis and the matched adjacent normal tissues in the PTC dataset from TCGA. (I) Real-time PCR analysis of MFSD4A-AS1 expression in our 48 ANT, 36 T1–2 PTC tissues without lymphatic metastasis and 38 T1–2 PTC tissues with lymphatic metastasis. GAPDH was used as endogenous control. n.s. indicates no significant difference. (J) MFSD4A-AS1 expression in 59 ANT, 164 T1-2 PTC tissues without lymphatic metastasis and 110 T1-2 PTC tissues with lymphatic metastasis in the PTC dataset from TCGA. n.s. indicates no significant difference.

To explore the lymph node metastasis-associated lncRNAs, we first investigated the RNA sequencing dataset of PTC from TCGA. As shown in Fig. 1A and Supplementary Fig. 1A, we found that lncRNA-MFSD4A-AS1 expression was increased in separate 503 PTC tissues, 59 paired PTC tissues compared with the adjacent normal tissues (ANT). In addition, 503 PTC was further sub-categorized into 357 classic PTC, 102 follicular variants of PTC, 36 tall cell variants of PTC and 8 other variants, including 4 diffuse sclerosing variants, 2 cribriform morular variants, 1 columnar cell variant and 1 papillary and follicular variant, and MFSD4A-AS1 level was differentially increased in classic, tall cell and other variants, but decreased in follicular variant compared with that in ANT (Fig. 1B). Consistently, MFSD4A-AS1 expression was demonstrated to be higher in our PTC without distant metastasis tissues than that in ANT (Fig. 1C). To further underscore the clinical significance of MFSD4A-AS1 in PTC with lymph node metastasis (TC-N1), we stratified PTC tissues based on lymph node metastatic status. First, we found that MFSD4A-AS1 was elevated in all TC-N1 tissues compared to PTC tissues without lymph node metastasis (TC-N0) by analyzing our 48 paired PTC tissues (Fig. 1D). Of note, MFSD4A-AS1 expression was specifically and significantly upregulated in TC-N1 tissues compared with that in ANT in our and TCGA datasets (Fig. 1E and F), but there was no statistical difference of MFSD4A-AS1 expression between TC-N0 and ANT (Fig. 1E and F), including in T1–2 and T3–4 subgroups, respectively (Supplementary Fig. 1B and C). Importantly, MFSD4A-AS1 was also specifically elevated in paired TC-N1 tissues compared with the matched ANT in our and TCGA datasets (Fig. 1G and H), and there was no statistical difference between paired TC-N0 tissues and ANT (Supplementary Fig. 1D and E). Even in the subgroup of T1–2 PTC, MFSD4A-AS1 was only upregulated in TC-N1 tissues (Fig. 1I and J) but did not show significant differential expression between TC-N0 and ANT (Fig. 1H and I). Collectively, our clinical findings in combination with the results of the PTC dataset from TCGA indicate that MFSD4A-AS1 may play a pivotal role in the lymphatic metastasis of PTC.

### MFSD4A-AS1 promotes lymphatic metastasis by promoting lymphangiogenesis-inducing and invasive abilities of PTC cells *in vivo*

**Figure 2:**
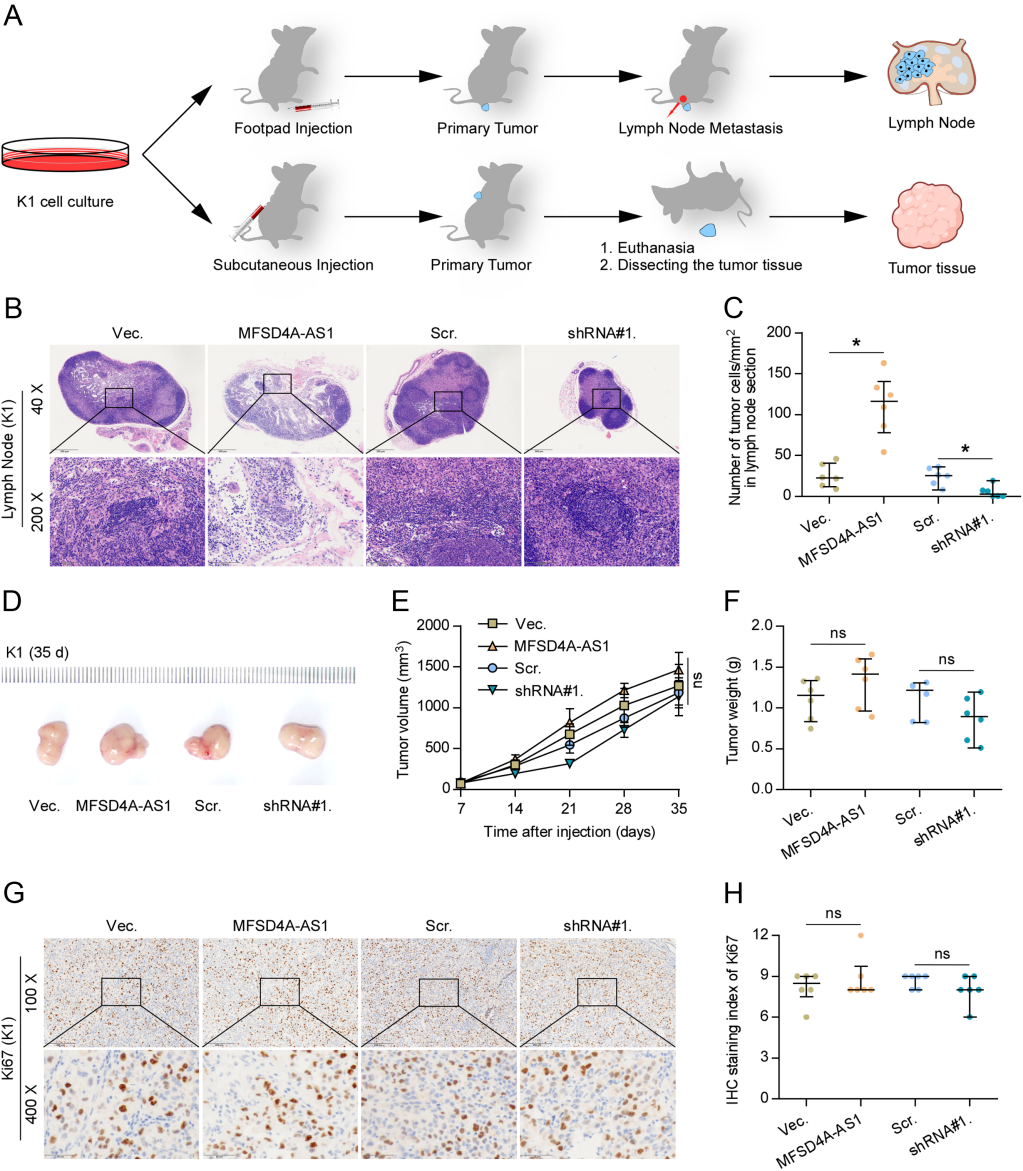
MFSD4A-AS1 promotes lymphatic metastasis of PTC cells *in vivo*. (A) Schematic model of lymphatic metastasis (upper panel) and s.c. injection (upper panel) model *in vivo*. (B) H & E staining analysis of tumors in lymph nodes from the indicated mice group. (C) The number of tumor cells in the tumor areas of lymph nodes from the indicated mice group. *P < 0.05. (D) The representative tumors formed by the indicated K1 cells in each mice group (*n* = 6, each group). (E) The effect of MFSD4A-AS1 on the tumor volumes in the indicated mice groups from the seventh day at 7 days interval after inoculation of 5 × 10^6^ cells. Data presented are the mean ± s.d. n.s. indicates no significant difference. (F) The effect of MFSD4A-AS1 on the tumor weights in the indicated mice groups after inoculation of 5 × 10^6^ cells. n.s. indicates no significant difference. (G) The representative images of Ki-67 staining in the tumor tissues from the indicated mice groups. (H) The Ki-67 staining scores in the tumor tissues from the indicated mice groups. n.s. indicates no significant difference.

To determine the role of MFSD4A-AS1 in lymphatic metastasis of PTC cells, we first examined the expression levels of MFSD4A-AS1 in a normal thyroid follicular epithelial cell line PTFE and seven thyroid cancer cell lines and found that MFSD4A-AS1 expression levels were higher to a different extent in thyroid cancer cells than that in PTFE cells (Supplementary Fig. 2A). We further endogenously knocked down MFSD4A-AS1 expression and stably overexpressed MFSD4A-AS1 in B-CPAP and K1 cells, because these two cell lines showed moderate levels of MFSD4A-AS1 compared to other thyroid cancer cell lines (Supplementary Fig. 2B), and the tumorigenic role of K1 in animal experiment *in vivo* was markedly superior to other PTC cell lines based on our previous experiments. Through the inguinal lymph node metastatic model, we first injected the indicated PTC cells into the surrounding foot pads tissues of the mice (Fig. 2A). Four weeks after cell inoculations, we found that overexpressing MFSD4A-AS1 dramatically increased the number of tumor cells in lymph nodes from the MFSD4A-AS1-overexpressing mice group compared with that in vector mice group (Fig. 2B and C). By contrast, silencing MFSD4A-AS1 reduced the number of tumor cells in lymph nodes derived from the MFSD4A-AS1-silencing mice group (Fig. 2B and C).

**Figure 3:**
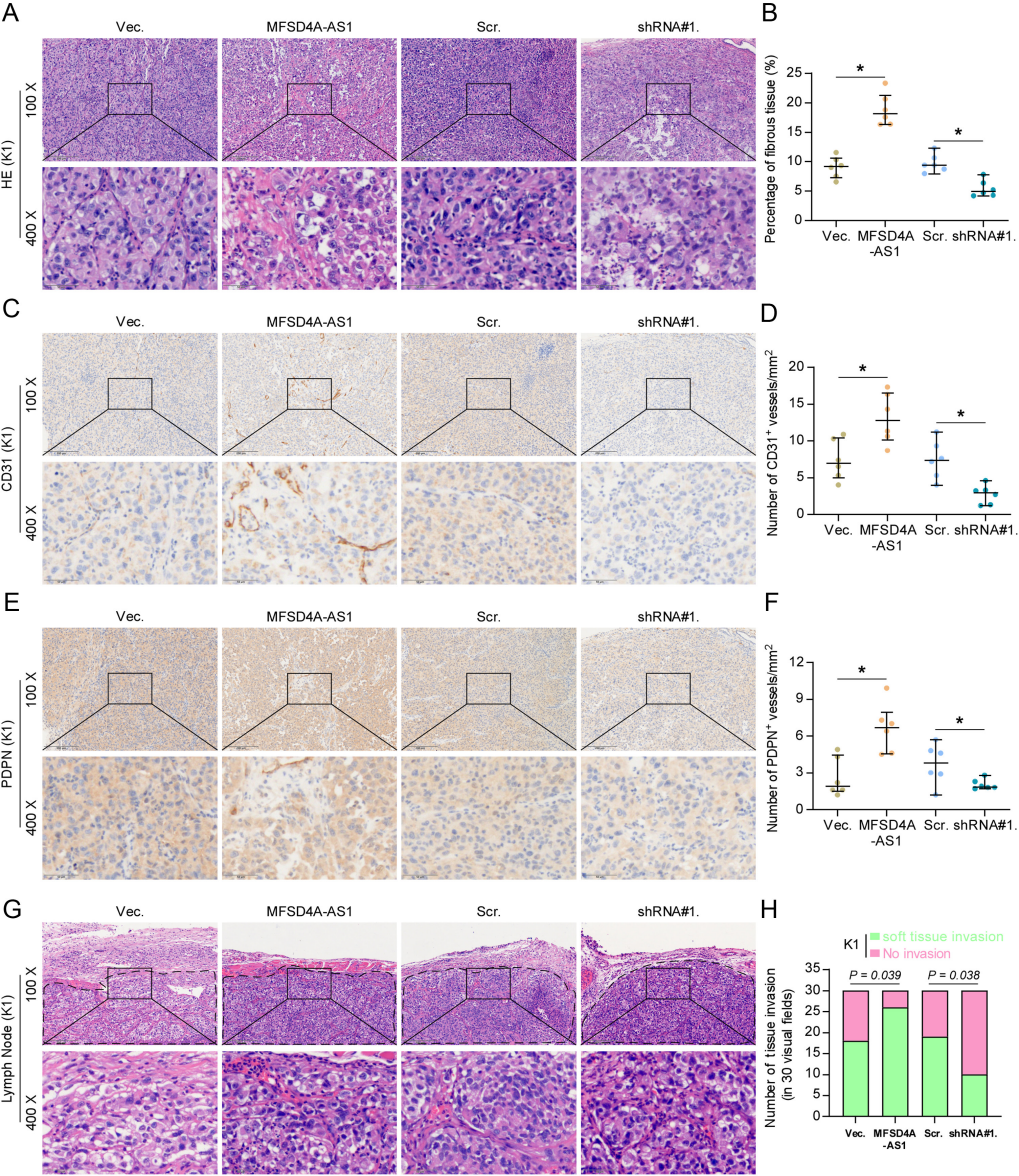
MFSD4A-AS1 promotes lymphangiogenesis and local invasion of PTC cells *in vivo*. (A and B) H & E staining analysis, and CD31 and PDPN staining in the tumor tissues from the indicated mice groups. *P < 0.05. (C and D) The CD31 staining scores in the tumor tissues from the indicated mice groups. *P < 0.05. (E and F) The PDPN staining scores in the tumor tissues from the indicated mice groups. *P < 0.05. (G) H & E staining analysis of the local invasive ability of PTC cells in the tumor tissues from the indicated mice groups. (H) The effect of MFSD4A-AS1 on muscle and fat infiltration of the adjacent tissues in the tumor tissues from the indicated mice groups. *P < 0.05.

To further investigate the biological function of MFSD4A-AS1 in lymphangiogenesis *in vivo*, the vector, MFSD4A-AS1-overexpressing, scramble and MFSD4A-AS1-silencing K1 cells were subcutaneously injected into the inguinal folds of the mice (Fig. 2A). As shown in Fig. 2D, E, F, G and H, neither tumor volume or weight nor Ki-67 staining index was affected by changed expression of MFSD4A-AS1 *in vivo*. Importantly, our results further revealed that upregulating MFSD4A-AS1 increased, while silencing MFSD4A-AS1 reduced the density of lymphatic or blood vessel (LBV) in tumor tissues, as demonstrated by the CD31+ and PDPN+ LBV per mm^2^ in tumor sections (Fig. 3A, B, C, D, E and F). Microscopic examination of tumor morphology showed that MFSD4A-AS1-overexpressing tumor tissues displayed more potent capabilities to penetrate into the surrounding muscle tissues; conversely, silencing MFSD4A-AS1 drastically inhibited the local invasive capability of tumors (Fig. 3G and H), suggesting the stimulatory effect of MFSD4A-AS1 on the invasive ability of PTC cells *in vivo*. Taken together, our results demonstrate that MFSD4A-AS1 promotes lymphatic metastasis of PTC cells *in vivo* by inducing the lymphangiogenic formation and enhancing the invasive capability of PTC cells.

### MFSD4A-AS1 enhances lymphangiogenesis of vascular endothelial cells and invasion and migration of PTC cells

**Figure 4:**
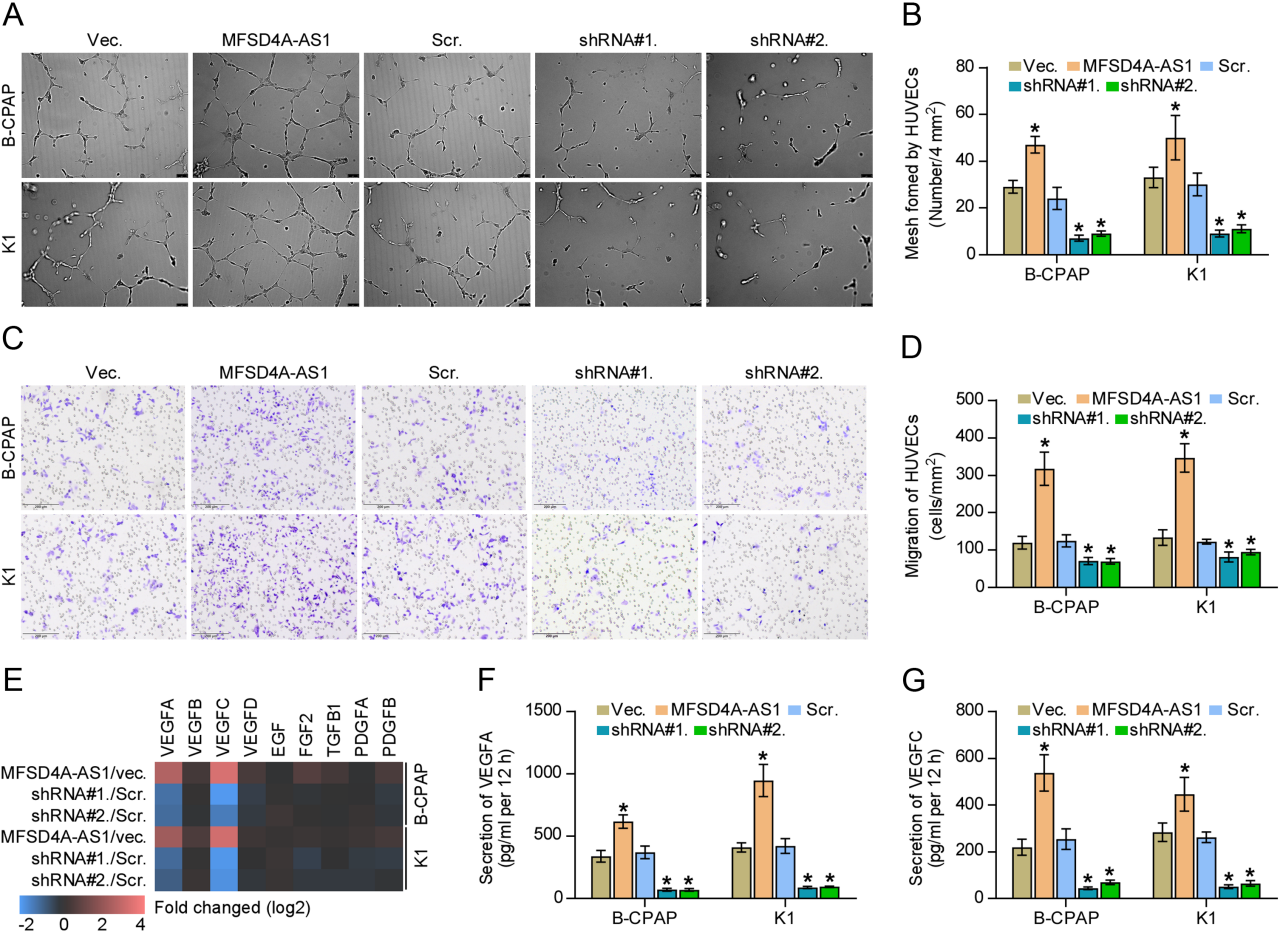
MFSD4A-AS1 enhances lymphangiogenesis of vascular endothelial cells of PTC cells. (A and B) The effect of changed expression of MFSD4A-AS1 in PTC cells on human umbilical vein endothelial cells (HUVECs) in tube formation assay. Each bar represents the mean values ± s.d. of three independent experiments. *P < 0.05. (C and D) The effect of changed expression of MFSD4A-AS1 in PTC cells on migration ability of HUVECs in Transwell assay. Each bar represents the mean values ± s.d. of three independent experiments. *P < 0.05. (E) Real-time PCR analysis of the effect of MFSD4A-AS1 on the expression of multiple proliferation and migration of vascular endothelial cells-associated (angiogenesis-associated) genes. Pseudo-color scale values were log_2_ transformed. Transcript levels were normalized by GAPDH expression. The experiment was independently performed three times. (F and G) The effect of MFSD4A-AS1 on secretion level of VEGFA and VEGFC in PTC cells by enzyme-linked immunosorbent assay (ELISA). Each bar represents the mean values ± s.d. of three independent experiments. *P < 0.05.

**Figure 5:**
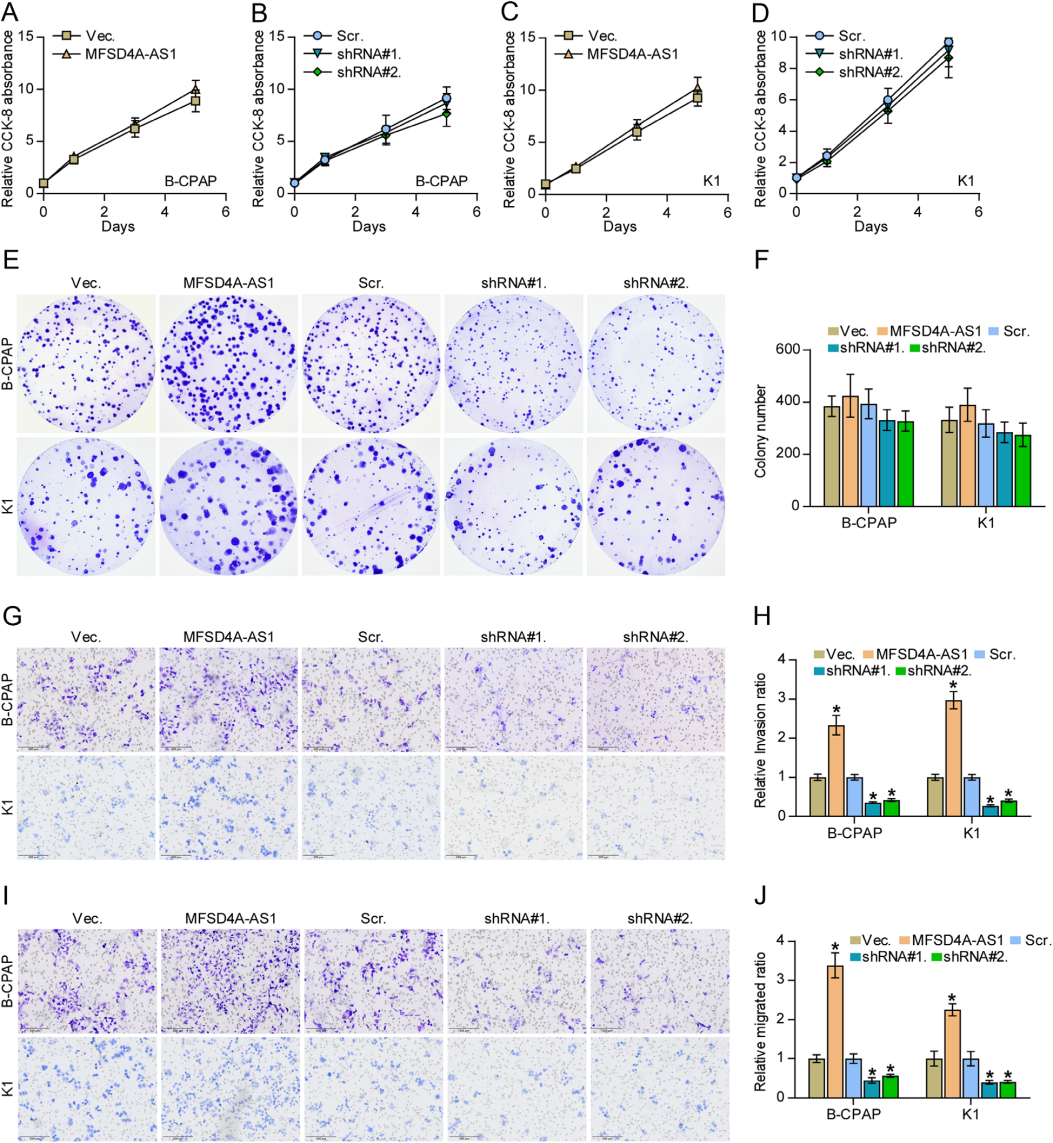
MFSD4A-AS1 promotes invasion and migration abilities but does not affect proliferation in PTC cells. (A–D) The effect of overexpression or silencing MFSD4A-AS1 on the cell growth in the indicated PTC cells by CCK-8 assay. (E and F) The effect of overexpression or silencing MFSD4A-AS1 on colony-formation ability of the indicated PTC cells by colony-formation assay. (G and H) The effect of overexpression or silencing MFSD4A-AS1 on invasion ability of PTC cells. Each bar represents the mean values ± s.d. of three independent experiments. *P < 0.05. (I and J) The effect of overexpression or silencing MFSD4A-AS1 on migration ability of PTC cells. Each bar represents the mean values ± s.d. of three independent experiments. *P < 0.05.

The biological function of MFSD4A-AS1 in lymphatic metastasis of PTC was further determined in a series of functional experiments *in vitro*. First, we performed tube formation and Transwell assays to investigate the effect of MFSD4A-AS1 on the proliferation and migration of vascular endothelial cells and found that the condition medium from MFSD4A-AS1-overexpressing PTC cells promoted mesh formation by HUVECs and the migration ability of HUVECs, whereas silencing MFSD4A-AS1 exhibited an opposite effect (Fig. 4A, B, C and D). In addition, upregulating MFSD4A-AS1 increased, while silencing MFSD4A-AS1 decreased expression of VEGFA and VEGFC (Fig. 4E). Notably, the secretion levels of both VEGFA and VEGFC were affected by the changed expression of MFSD4A-AS1 (Fig. 4F and G). Consistent with the findings *in vivo* (Fig. 2D, E, F, G and H), the changed expression of MFSD4A-AS1 had no significant effect on the cell growth of PTC cells (Fig. 5A, B, C, D, E and F). In addition, upregulating MFSD4A-AS1 enhanced, while silencing MFSD4A-AS1 inhibited the invasion and migration capabilities of PTC cells (Fig. 5G, H, I and J). Therefore, our results reveal that MFSD4A-AS1 promotes lymphangiogenesis-inducing, invasive and migration capabilities of PTC cells *in vitro*.

### MFSD4A-AS1 functions as a competing endogenous RNA by sponging miR-30c-2-3p, miR-145-3p and miR-139-5p

**Figure 6:**
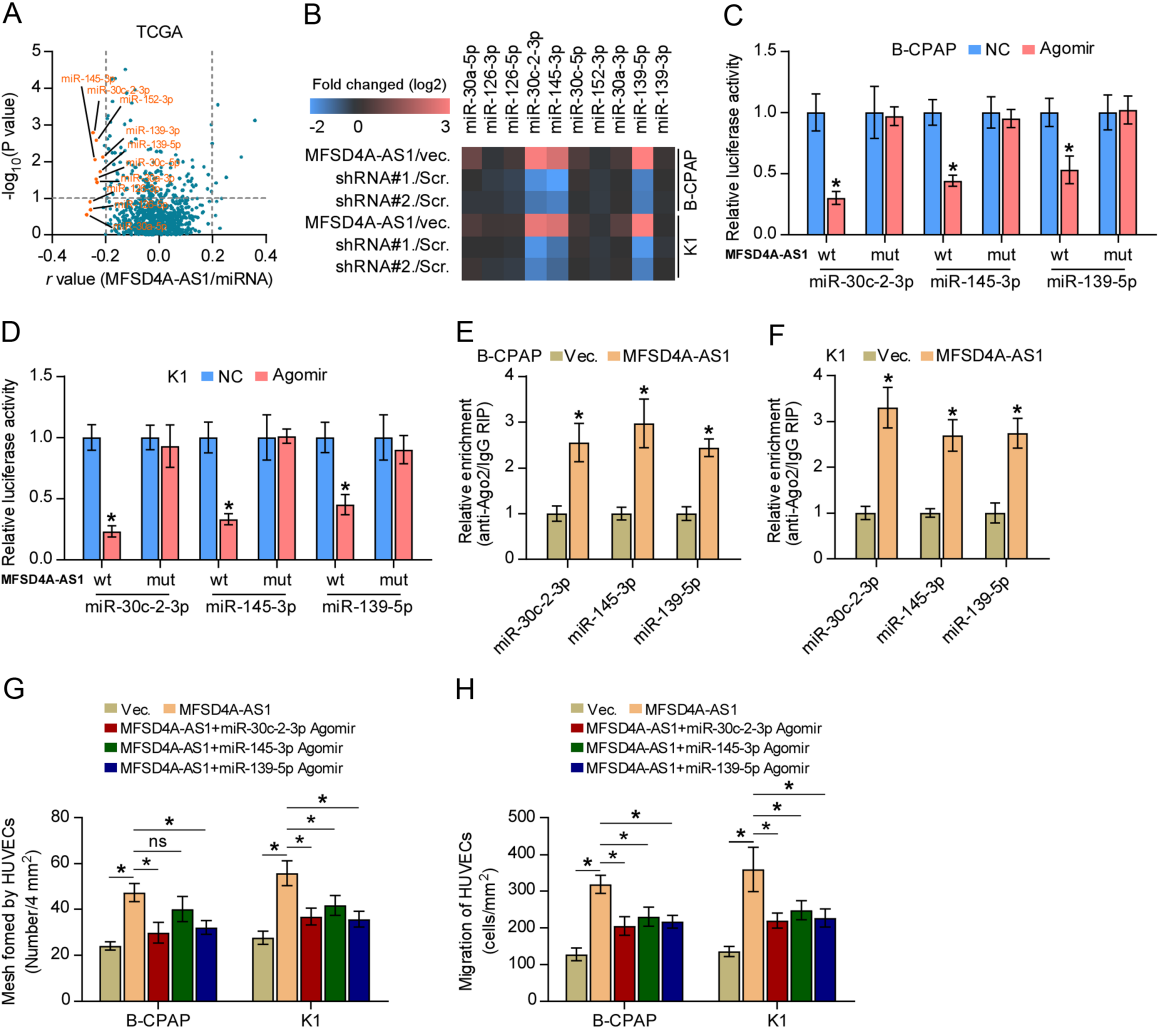
MFSD4A-AS1 sponges miR-30c-2-3p, miR-145-3p and miR-139-5p. (A) Volcano plot analyzed the clinical correlation of MFSD4A-AS1 with all reported miRNAs in the PTC dataset from TCGA. The orange colors represent significantly and negatively correlated miRNAs with fold change > 2 and *r* value < −0.2. (B) Real-time PCR analysis of the effect of MFSD4A-AS1 on expression of miR-30a-5p, miR-126-3p, miR-126-5p, miR-30c-2-3p, miR-145-3p, miR-30c-5p, miR-152-3p, miR-30a-3p, miR-139-5p and miR-139-3p. Pseudo-color scale values were log_2_ transformed. Transcript levels were normalized by U6 expression. The experiment was independently performed three times. (C and D) The effect of miR-30c-2-3p, miR-145-3p and miR-139-5p on the luciferase activity of wild-type or mutant MFSD4A-AS1 in the indicated cells. Error bars represent the mean ± s.d. of three independent experiments. *P < 0.05. (E and F) RNA immunoprecipitation (RIP) assay showing the association among miR-30c-2-3p, miR-145-3p and miR-139-5p, and MFSD4A-AS1 transcripts in PTC cells. Pulldown of IgG antibody served as the negative control *P < 0.05. (G) The mediating effect of changed expression of miR-139-5p, miR-145-3p and miR-30c-2-3p in PTC cells on human umbilical vein endothelial cells (HUVECs) in tube formation assay. Each bar represents the mean values ± s.d. of three independent experiments. *P < 0.05. (H) The mediating effect of changed expression of miR-139-5p, miR-145-3p and miR-30c-2-3p in PTC cells on migration ability of HUVECs in the Transwell assay. Each bar represents the mean values ± s.d. of three independent experiments. *P < 0.05.

Numerous studies have shown that lncRNAs can serve as competitive endogenous RNAs (ceRNAs) to regulate downstream mRNAs expression by sponging miRNAs (Karreth *et al.* 2011, Tay *et al.* 2011). Then, the miRNAs binding to MFSD4A-AS1 were further explored by analyzing the correlation of MFSD4A-AS1 with all reported miRNAs in the PTC dataset from TCGA. As shown in Fig. 6A, the expression levels of ten miRNAs, including miR-30a-5p, miR-126-3p, miR-126-5p, miR-30c-2-3p, miR-145-3p, miR-30c-5p, miR-152-3p, miR-30a-3p, miR-139-5p and miR-139-3p, were significantly inversely correlated with MFSD4A-AS1 expression. Real-time PCR analysis further demonstrated that only miR-30c-2-3p, miR-145-3p and miR-139-5p were significantly affected by the changed expression of MFSD4A-AS1 (Fig. 6B). Notably, miR-139-5p was reported to serve as a prognostic marker in PTC patients and functioned as a tumor-suppressive miRNA in PTC (Montero-Conde *et al.* 2020). Importantly, miR-30c-2-3p was found to be positively correlated with lymph node metastasis of PTC (Saiselet *et al.* 2015). Through analyzing publicly available algorithms, we found that miR-30c-2-3p, miR-145-3p and miR-139-5p had the potential recognition sequences on MFSD4A-AS1 (Supplementary Fig. 3A, B and C). Luciferase assay demonstrated that miR-30c-2-3p, miR-145-3p and miR-139-5p agomir reduced the 3'UTR reporter activity of MFSD4A-AS1, but not of the mutant 3'UTR of MFSD4A-AS1 (Fig. 6C and D). RNA immunoprecipitation (RIP) assays demonstrated direct associations of miR-30c-2-3p, miR-145-3p and miR-139-5p with the transcripts of MFSD4A-AS1 (Fig. 6E and F). Importantly, upregulating miR-30c-2-3p, miR-145-3p and miR-139-5p differentially attenuated the pro-mesh formation and migration ability of MFSD4A-AS1 overexpression in PTC cells (Fig. 6G and H). Thus, these results indicate that MFSD4A-AS1 sponge miR-30c-2-3p, miR-145-3p and miR-139-5p as a ceRNA to inhibit their expression.

### MFSD4A-AS1 elevates VEGFA and VEGFC dependent on different downstream miRNAs

By analyzing multiple publically available algorithms, we found that VEGFC may be the potential targets of miR-30c-2-3p and miR-145-3p, and VEGFA be the target of miR-139-5p (Supplementary Fig. 4A). Real-time PCR analysis demonstrated that upregulating miR-30c-2-3p and miR-145-3p differentially reduced the mRNA levels of VEGFC, and only overexpression of miR-139-5p decreased VEGFA expression in PTC cells (Supplementary Fig. 4B and C); conversely, silencing miR-30c-2-3p and miR-145-3p elevated VEGFC expression, and downregulating miR-139-5p enhanced VEGFA expression (Supplementary Fig. 4B and C). Consistently, ELISA assay further showed that upregulating miR-30c-2-3p and miR-145-3p decreased while silencing miR-30c-2-3p and miR-145-3p increased the secretion levels of VEGFC; upregulating miR-139-5p reduced while silencing miR-139-5p increased the secretion levels of VEGFA (Supplementary Fig. 4D and E). RIP assay demonstrated direct correlations of miR-30c-2-3p and miR-145-3p with the transcript of VEGFC and miR-139-5p with VEGFA transcript (Supplementary Fig. 4F and G). Luciferase assay further revealed that upregulating miR-30c-2-3p and miR-145-3p decreased, whereas silencing miR-30c-2-3p and miR-145-3p enhanced the 3'UTR reporter activity of VEGFC, and changed expression of miR-139-5p had negative effect on the 3'UTR reporter activity of VEGFA, but not of the mutant ones (Supplementary Fig. 4H and I). Further investigation showed that only miR-139-5p reduced VEGFA secretion in MFSD4A-AS1-overexpressing PTC cells (Supplementary Fig. 4J). However, miR-30c-2-3p and miR-145-3p but not miR-139-5p could reverse the stimulatory effect of MFSD4A-AS1 overexpression on secretion levels of VEGFC (Supplementary Fig. 4K). Therefore, these findings demonstrate that MFSD4A-AS1 enhances VEGFA and VEGFC expression and promotes their secretion by sponging miR-139-5p, and miR-30c-2-3p and miR-145-3p, respectively, to disrupt the inhibitory effects of these three miRNAs on VEGFA and VEGFC.

### MFSD4A-AS1 promotes lymphatic metastasis and proliferation and migration of HUVECs by upregulating VEGFA and VEGFC

**Figure 7:**
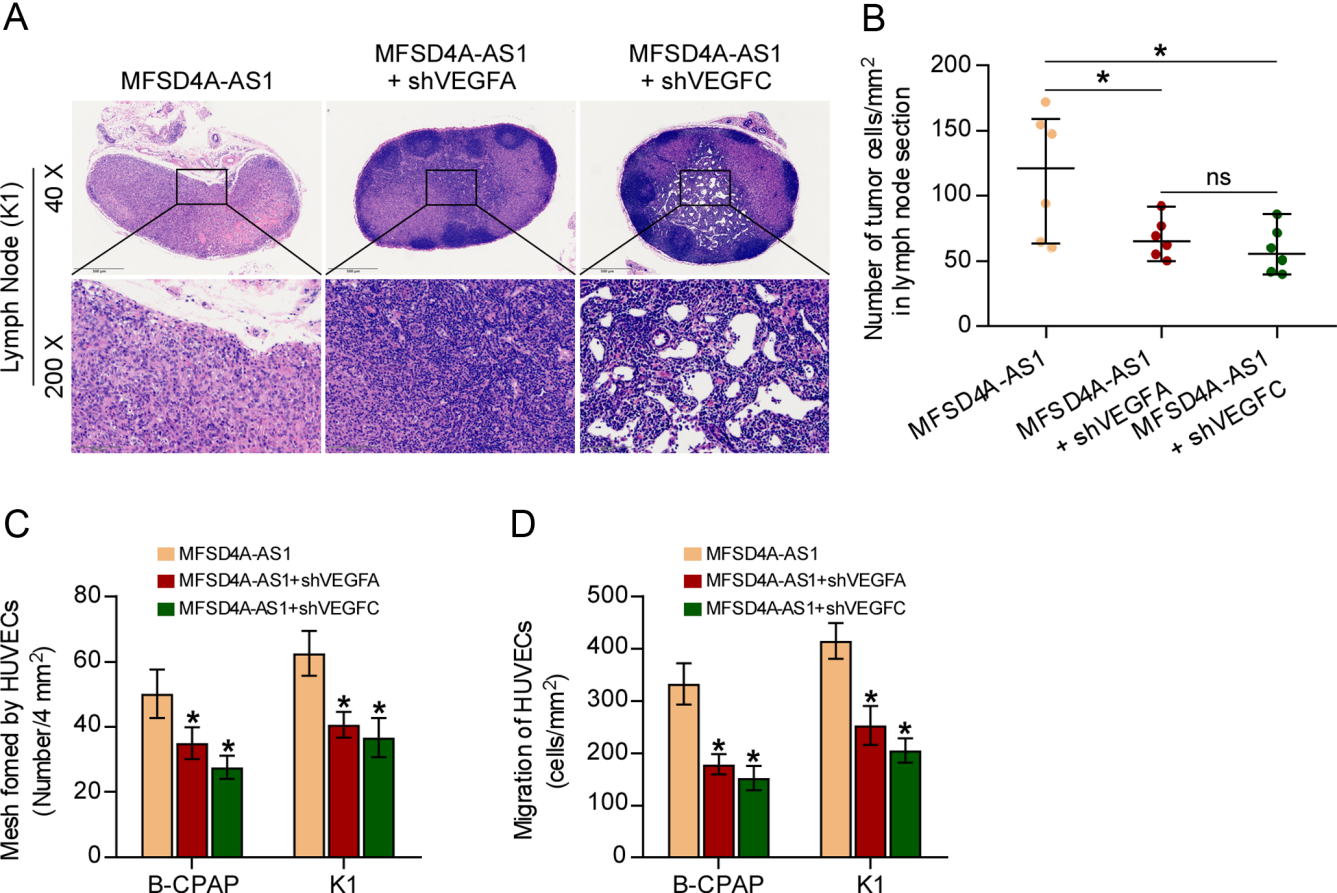
MFSD4A-AS1 promotes lymphatic metastasis and proliferation and migration of HUVECs by upregulating VEGFA and VEGFC. (A) H & E staining analysis of tumors in lymph nodes from the indicated mice group. (B) The number of tumor cells in the tumor areas of lymph nodes from the indicated mice group. *P < 0.05. (C) The effect of silencing VEGFA or VEGFC in MFSD4A-AS1 overexpression PTC cells on tube formation ability of HUVECs in tube formation assay. Each bar represents the mean values ± s.d. of three independent experiments. *P < 0.05. (D) The effect of silencing VEGFA or VEGFC in MFSD4A-AS1 overexpression PTC cells on migration ability of HUVECs in the Transwell assay. Each bar represents the mean values ± s.d. of three independent experiments. *P < 0.05.

We further investigated whether VEGFA and VEGFC have an influence on the overexpression of MFSD4A-AS1-induced lymphatic metastasis and proliferation and migration of HUVECs. First, animal experiments *in vivo* showed that separately silencing VEGFA or VEGFC significantly reduced the number of tumor cells in lymph nodes from the MFSD4A-AS1-overexpressing mice group (Fig. 7A and B). Consistently, HUVECs tube formation and Transwell assays indicated that silencing VEGFA or VEGFC differentially attenuated the pro-mesh formation and migration ability of MFSD4A-AS1 overexpression in PTC cells (Fig. 7C and D). Collectively, our results demonstrated that VEGFA and VEGFC mediate the role of MFSD4A-AS1 overexpression in promoting lymphatic metastasis and proliferation and migration of HUVECs *in vivo and in vitro* in PTC.

### MFSD4A-AS1 promotes lymphatic metastasis, proliferation and migration of HUVECs by partially activating TGF-β signaling

**Figure 8:**
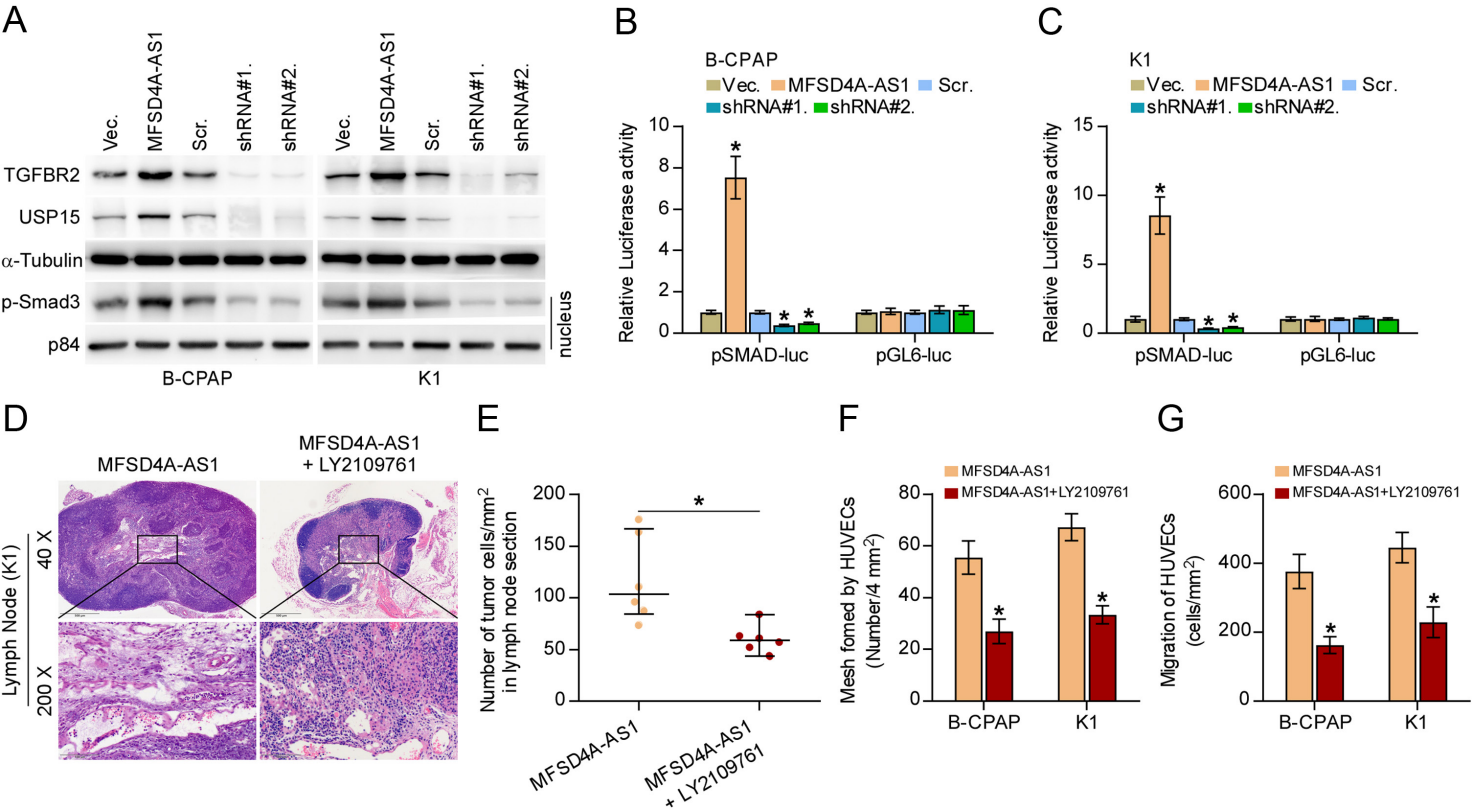
MFSD4A-AS1 promotes lymphatic metastasis, invasion and migration of PTC cells partially by activating TGF-β signaling. (A) Western blotting analysis of the effect of overexpression or silencing MFSD4A-AS1 on TGFBR2, USP15 and nuclear translocation of phosphorylated Smad3 (p-Smad3) in the indicated PTC cells. α-Tubulin and p84 served as the cytoplasmic and nuclear loading control, respectively. (B and C) The effect of overexpression or silencing MFSD4A-AS1 on TGF-β/Smad-responsive luciferase reporter in the indicated cells. Error bars represent the mean ± s.d. of three independent experiments. *P < 0.05. (D) H & E staining analysis of tumors in lymph nodes from the indicated mice group. (E) The number of tumor cells in the tumor areas of lymph nodes from the indicated mice group. *P < 0.05. (F) The effect of TGF-β inhibitor LY2109761 in MFSD4A-AS1 overexpression PTC cells on tube formation ability of HUVECs in tube formation assay. Each bar represents the mean values ± s.d. of three independent experiments. *P < 0.05. (G) The effect of TGF-β inhibitor LY2109761 in MFSD4A-AS1 overexpression PTC cells on migration ability of HUVECs in the Transwell assay. Each bar represents the mean values ± s.d. of three independent experiments. *P < 0.05.

Fang and colleagues have reported that miR-30c-2-3p targeted TGFBR2 and USP15 in non-small cell lung cancer (NSCLC) cells, leading to the inactivation of TGF-β signaling (Fang *et al.* 2019). Of note, accumulating studies have reported that constitutive activation of TGF-β signaling plays a crucial role in lymphatic metastasis of tumors by inducing VEGFC expression (Liu *et al.* 2014, Huang *et al.* 2017, Shu *et al.* 2017), including PTC (He *et al.* 2018*a*). Importantly, inhibition of TGF-β signaling activity effectively suppressed lymphatic metastasis of NSCLC (Salvo *et al.* 2014), suggesting the potential value of therapeutic strategy targeting TGF-β signaling in cancer. Therefore, we surmised that TGF-β signaling might mediate the role of MFSD4A-AS1 in promoting lymphangiogenesis of HUVECs given the finding that MFSD4A-AS1 inhibited miR-30c-2-3p expression in PTC cells (Fig. 6B), as well as the partial role of miR-30c-2-3p in reversing the effect of MFSD4A-AS1 overexpression on lymphangiogenesis of HUVECs. First, our results demonstrated that upregulating MFSD4A-AS1 not only elevated TGFBR2 and USP15 expression (Fig. 8A) but also increased nuclear pSMAD3 expression and transcriptional activity of the TGF-β/Smad-responsive luciferase reporter plasmid CAGA12 (Fig. 8A, B and C). Conversely, silencing MFSD4A-AS1 yielded an opposite effect (Fig. 8A, B and C). Of note, only miR-30c-2-3p agomir significantly reversed the effect of MFSD4A-AS1 overexpression on increased expression of TGFBR2 and USP15, as well as activation of TGF-β signaling (Supplementary Fig. 5A, B and C), and neither miR-145-3p nor miR-139-5p had an effect on the activity of TGF-β signaling (Supplementary Fig. 5D, E and F). Furthermore, either silencing TGFBR2 or USP15 or blocking activity of TGF-β signaling using LY2109761, a novel selective TGF-β receptor type I/II dual inhibitor (Melisi *et al.* 2008), differentially abrogated the stimulatory effect of MFSD4A-AS1 on invasive and migration abilities of PTC cells (Supplementary Fig. 6A and B). In turn, TβR1 TD, a constitutively active mutant TGFBRI plasmid containing a substitution of threonine 204 with aspartic acid (T204D) that leads to constitutive activation of TGF-β signaling in the absence of TGF-β stimulation (53, 54), significantly reversed the inhibitory effect of silencing MFSD4A-AS1 on invasive and migration capabilities of PTC cells (Supplementary Fig. 6C and D). Importantly, LY2109761 dramatically abrogated the pro-lymphatic metastasis role of MFSD4A-AS1-overexpression *in vivo* (Fig. 8D and E), and the pro-mesh formation of and migration ability of HUVECs *in vitro* in PTC cells (Fig. 8F and G). Collectively, our results showed that MFSD4A-AS1 promotes lymphatic metastasis, invasion and migration of PTC cells at least partially by activating TGF-β signaling.

## Discussion

The critical findings of the current study present novel dissection for the first time, to the best of our knowledge, into the crucial role of MFSD4A-AS1 as a ceRNA in lymphatic metastasis of PTC. Here, we reported that MFSD4A-AS1 was remarkably and specifically upregulated in PTC tissues with lymph node metastasis. Functional experiments revealed that upregulating MFSD4A-AS1 promoted, while silencing MFSD4A-AS1 suppressed lymphatic metastasis of PTC cells *in vivo*, and proliferation and migration of HUVECs and invasion and migration of PTC cells *in vitro*. Our results further clarified that upregulating MFSD4A-AS1 promoted lymphatic metastasis of PTC by sequestering miR-30c-2-3p, miR-145-3p and miR-139-5p to disrupt the miRNAs-mediated inhibition of VEGFA and VEGFC and inactivation of TGF-β signaling. Collectively, our results determine the oncogenic role and underlying mechanism of MFSD4A-AS1 in lymphatic metastasis of PTC.

Numerous studies have demonstrated that the dysreuglation or aberrant expression of lncRNAs significantly contributed to lymphatic metastasis in various cancer types (Chen *et al.* 2018, He *et al.* 2018*b*). In the context of PTC, lncRNAs have been extensively documented to be able to function as oncogenic or tumor-suppressive factors to promote progression and metastasis of PTC (Gou *et al.* 2018, Esposito *et al.* 2019, Zhuang *et al.* 2019). The prime issue of PTC is its high avidity to metastasize to lymph node in which lncRNAs play an important role (Xia *et al.* 2017, Feng *et al.* 2019). Our previous study has shown that lncRNA MAPK8IP1P2 inhibited lymphatic metastasis of PTC by activating Hippo signaling via sponging miR-146b-3p (Liu *et al.* 2020). However, the clinical significance and functional role of MFSD4A-AS1 in PTC, especially in lymph node metastatic PTC, remain blank. In this study, our results demonstrated that there was no statistical difference in MFSD4A-AS1 expression between PTC tissues without lymph node metastasis and ANT, including in the T1–2 and T3–4 subgroups. Importantly, MFSD4A-AS1 was specifically and significantly upregulated in PTC tissues with lymph node metastasis compared with that in ANT and PTC tissues without lymph node metastasis. Gain- and loss-of-function assays demonstrated that upregulating MFSD4A-AS1 promoted, while silencing MFSD4A-AS1 suppressed proliferation and migration of HUVECs and invasion and migration of PTC cells *in vitro*, and lymphatic metastasis of PTC cells *in vivo*. Based on these findings, our results for the first time provide experimental evidence with regard to the clinical significance of MFSD4A-AS1 in PTC, particularly shedding light on the critical role of MFSD4A-AS1 in lymphatic metastasis of PTC.

As a class of versatile ncRNA, lncRNAs have been extensively validated to exert their biological functions depending on the subcellular localization of the lncRNAs, such as interacting with DNA within the nucleus as enhancers to regulate the target genes transcription of, as scaffold molecules to modulate protein/gene or protein/protein interactions (Ponting *et al.* 2009, Salehi *et al.* 2017). However, more and more attention focusing on the role of lncRNAs as ceRNAs by sponging target miRNAs to disrupt the miRNAs-mediated degradation of target genes has been made recently in cancer progression and metastasis (Karreth *et al.* 2011, Tay *et al.* 2011, Xu *et al.* 2019, Lang *et al.* 2020). Importantly, miRNA-mediated lncRNA/mRNA crosstalk plays a critical role in the development and metastasis of thyroid cancer (Credendino *et al.* 2019, Liu *et al.* 2019, Yao *et al.* 2019), including lymphatic metastasis (Feng *et al.* 2019, Zhuang *et al.* 2019, Li *et al.* 2020). In the current study, our results found that MFSD4A-AS1 functioned as ceRNA to sequester miR-30c-2-3p, miR-145-3p and miR-139-5p to disrupt the miRNA-mediated inhibition of VEGFA and VEGFC on the one hand; on the other hand, MFSD4A-AS1 further activated TGF-β signaling by sponging miR-30c-2-3p to relieve the repressive effect of miR-30c-2-3p on TGFBR2 and USP15, which synergistically promoted lymphangiogenesis and lymphatic metastasis of PTC. Therefore, our findings unravel novel dual mechanisms by which MFSD4A-AS1 promoted lymphatic metastasis of PTC.

TGF-β signaling has been widely reported to be implicated in multiple biological processes, such as cell proliferation and differentiation and bone remodeling (Mohammad *et al.* 2009). Interestingly, TGF-β signaling exerts a complex and even paradoxical function in cancer scenario depending on the developmental stage of tumor: TGF-β signaling functions as a tumor suppressor to inhibit cell growth in early stages; whereas in later stages, TGF-β signaling plays an oncogenic role in promoting invasion and metastasis of tumor cells (Pickup *et al.* 2013). Furthermore, activation of TGF-β signaling has been demonstrated to expedite lymphatic metastasis of tumors via varying mechanisms (Liu *et al.* 2014, Huang *et al.* 2017, Shu *et al.* 2017), where TGF-β-induced dramatical upregulation of VEGFC in tumor cells was regarded as the primary contributor (Liu *et al.* 2014). Notably, a fundamental study from He *et al.* has reported that SLC35F2 promoted the proliferation, migration and lymphatic metastasis of PTC by activating TGF-β signaling (He *et al.* 2018*a*), elucidating the critical role of TGF-β signaling in lymphatic metastasis of thyroid cancer. Importantly, inhibition of TGF-β signaling activity effectively suppressed lymphatic metastasis of NSCLC (Salvo *et al.* 2014), implying that therapeutic strategy targeting TGF-β signaling holds favorable prospects for the treatment of lymphatic metastasis of cancer. In this study, our results demonstrated that MFSD4A-AS1 upregulated TGFBR2 and USP15, and activated TGF-β signaling by sponging miR-30c-2-3p. Importantly, reconstituting activity of TGF-β signaling using transfecting TβR1 TD dramatically reversed the inhibitory effect of silencing MFSD4A-AS1 on invasion and migration of PTC cells. Taken together, our results suggested that therapeutic avenue by inhibition of MFSD4A-AS1 holds a promising efficacy in lymphatic metastasis of PTC. However, more solid conclusion regarding the therapeutic efficacy of MFSD4A-AS1 inhibition in lymphatic metastasis of PTC is definitely worth further investigation through a series of animal and clinical trials.

In summary, our results demonstrate that MFSD4A-AS1 promotes lymphangiogenesis and lymphatic metastasis of PTC by sequestering miR-30c-2-3p, miR-145-3p and miR-139-5p to disrupt the miRNAs-mediated both inhibition of VEGFA and VEGFC expression and inactivation of TGF-β signaling. An in-depth understanding of the functional role and underlying mechanism by which MFSD4A-AS1 promotes lymphatic metastasis of PTC will facilitate to clarify the pathogenesis of lymphatic metastasis of PTC.

## Supplementary Material

Supplemental Figure 1. (A) MFSD4A-AS1 expression in 59 paired PTC tissues and the matched ANT in the thyroid cancer dataset from TCGA. (B) Real-time PCR analysis of MFSD4A-AS1 expression in our 48 ANT, 36 T1-2 PTC tissues without lymphatic metastasis, and 11 T3-4 PTC tissues without lymphatic metastasis, GAPDH was used as endogenous controls. n.s. indicates no significant difference. (C) MFSD4A-AS1 expression in 59 ANT, 164 T1-2 PTC tissues without lymphatic metastasis, and 66 T3-4 PTC tissues without lymphatic metastasis in the thyroid cancer dataset from TCGA. n.s. indicates no significant difference. (D) Real-time PCR analysis of MFSD4A-AS1 expression in our 16 paired PTC tissues without lymphatic metastasis and the matched adjacent normal tissues. (E) MFSD4A-AS1 expression in 30 paired PTC tissues without lymphatic metastasis and the matched adjacent normal tissues in the PTC dataset from TCGA.

Supplemental Figure 2. (A) Real-time PCR analysis of MFSD4A-AS1 expression in 7 thyroid cancer cells, including 3 PTC cell lines, B-CPAP, KTC-1 and K1, and 4 anaplastic thyroid cancer (ATC) cell lines (BHT-101, CAL-62, KMH-2 and 8305C) and a normal thyroid follicular epithelial cell line PTFE. GAPDH was used as endogenous controls. *P<0.05. (B) MFSD4A-AS1 expression in the vector, MFSD4A-AS1 overexpression, scramble, MFSD4A-AS1 shRNA#1 and MFSD4A-AS1 shRNA#2 PTC cells using real-time PCR. Transcript levels were normalized by GAPDH expression. *P < 0.05.

Supplemental Figure 3. (A-C) Predicted recognition sites of miR-139-5p (a), miR-145-3p (b), and miR-30c-2-3p (c) on MFSD4A-AS1.

Supplemental Figure 4. MFSD4A-AS1 elevates VEGFA and VEGFC dependent on different downstream miRNAs. (A) Predicted miR-139-5p, miR-145-3p, and miR-30c-2-3p targeting sequences and mutant sequences in 3'UTR s of VEGFA and VEGFC. (B and C) Real-time PCR analysis of the effect of miR-139-5p, miR-145-3p, and miR-30c-2-3p on mRNA expression of VEGFA and VEGFC in the indicated PTC cells. GAPDH was used as endogenous controls. (D and E) The effect of miR-30c-2-3p, miR-145-3p and miR-139-5p on secretion level of VEGFA (D) and VEGFC (E) in PTC cells by ELISA. Each bar represents the mean values ± SD of three independent experiments. *P < 0.05. (F and G) RIP assay showing the association among miR-30c-2-3p, miR-145-3p and miR-139-5p, and VEGFA and VEGFC transcripts in PTC cells. Pulldown of IgG antibody served as the negative control *P < 0.05. (H and I) The effect of miR-139-5p, miR-145-3p, and miR-30c-2-3p on the luciferase activity of wild-type or mutant VEGFA and VEGFC in the indicated cells. Error bars represent the mean ± S.D. of three independent experiments. *P < 0.05. (J and K) The effect of miR-30c-2-3p, miR-145-3p and miR-139-5p on secretion level of VEGFA (J) and VEGFC (K) in PTC cells by ELISA. Each bar represents the mean values ± SD of three independent experiments. *P < 0.05. n.s. indicates no significant difference.

Supplemental Figure 5. (A) Western blotting analysis of the effect of miR-30c-2-3p on TGFBR2, USP15 and nuclear translocation of phophorylated Smad3 (p-Smad3) in the indicated PTC cells. α-Tubulin and p84 were served as the cytoplasmic and nuclear loading control respectively. (B and C) The effect of miR-30c-2-3p on TGF-β/Smad-responsive luciferase reporter in the indicated cells. Error bars represent the mean ± S.D. of three independent experiments. *P < 0.05. (D) Western blotting analysis of the effect of miR-139-5p or miR-145-3p on TGFBR2, USP15 and nuclear translocation of phophorylated Smad3 (p-Smad3) in the indicated PTC cells. α-Tubulin and p84 were served as the cytoplasmic and nuclear loading control respectively. (E and F) The effect of miR-139-5p or miR-145-3p on TGF-β/Smad-responsive luciferase reporter in the indicated cells. Error bars represent the mean ± S.D. of three independent experiments. *P < 0.05.

Supplemental Figure 6. (A and B) The effect of silencing TGFBR2, USP15 or LY2109761 on migration (A) and invasion (B) abilities of MFSD4A-AS1-overexpressing PTC cells. Each bar represents the mean values ± SD of three independent experiments. *P < 0.05. (C and D) The effect of TβR1 TD, a constitutively active mutant TGFBRI plasmid, on migration (C) and invasion (D) abilities of MFSD4A-AS1-downexpressing PTC cells. Each bar represents the mean values ± SD of three independent experiments. *P < 0.05.

Supplementary Material

Supplementary Data

## Declaration of interest

The authors declare no conflict of interest.

## Funding

This study was funded by the National Natural Science Foundation of China http://dx.doi.org/10.13039/501100001809 (grant number 81972499), the Department of Science and Technology of Jilin Province http://dx.doi.org/10.13039/501100011789, China (grant number 20200201181JC), the Finance Department of Jilin Province, China (grant number 2020SCZ51), and Jilin University http://dx.doi.org/10.13039/501100004032, China (grant number 2020B12).

## Author contribution statement

Liu Xiaoli and Sun Hui conceived the project and drafted the manuscript. Liu Xiaoli, Chunhai Zhang and Xiaomiao Wang conducted the experiments and contributed to the analysis of data. Can Cui and Hanwen Cui performed the animal experiments. Baishu Zhu and Anqi Chen analyzed the informatics data. Lu Zhang and Jingwei Xin performed IHC and the analysis of data. Qingfeng Fu contributed to the cell biology and molecular biology experiments. Liu Xiaoli and Gianlorenzo Dionigi edited and revised the manuscript. All authors discussed the results and approved the manuscript.
