# Supplementary figures and images for: Long non-coding RNA MFSD4A-AS1 promotes lymphangiogenesis and lymphatic metastasis of papillary thyroid cancer
Source: Endocr Relat Cancer. 2023 Feb 8;30(3):e220221. doi: 10.1530/ERC-22-0221 (PMC9986400; doi:10.1530/ERC-22-0221)

Supplement Figure 1

**A**

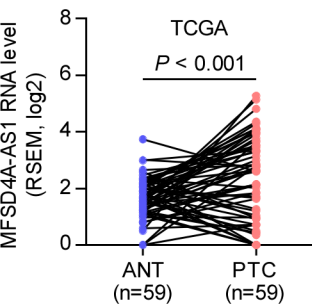

**B**

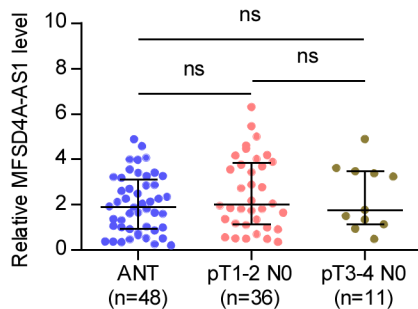

**C**

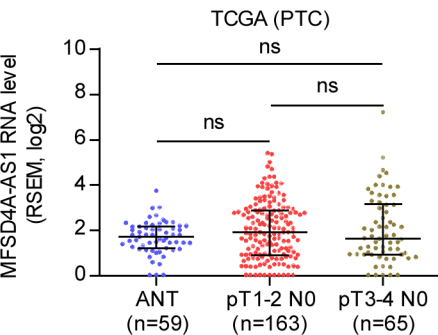

**D**

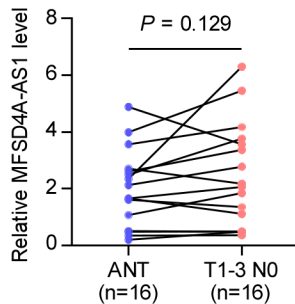

**E**

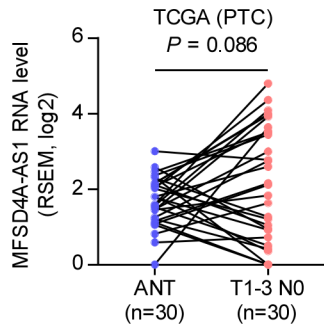

Supplement: Supplemental Figure 1. (A) MFSD4A-AS1 expression in 59 paired PTC tissues and the matched ANT in the thyroid cancer dataset from TCGA. (B) Real-time PCR analysis of MFSD4A-AS1 expression in our 48 ANT, 36 T1-2 PTC tissues without lymphatic metastasis, and 11 T3-4 PTC tissues without lymphatic metast [file supplementary_figure_1.pdf]

# Supplement Figure 2

**A**

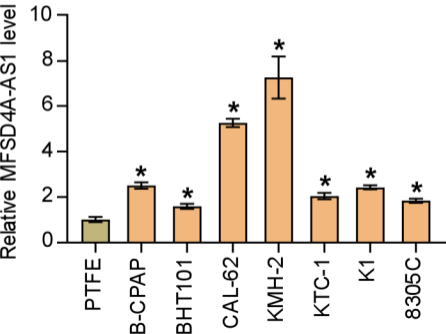

**B**

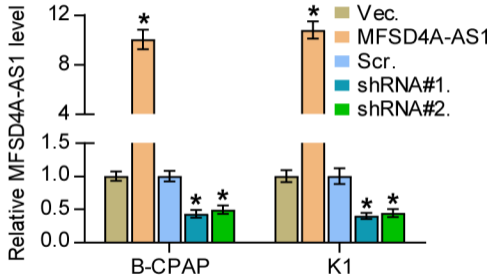

Supplement: Supplemental Figure 2. (A) Real-time PCR analysis of MFSD4A-AS1 expression in 7 thyroid cancer cells, including 3 PTC cell lines, B-CPAP, KTC-1 and K1, and 4 anaplastic thyroid cancer (ATC) cell lines (BHT-101, CAL-62, KMH-2 and 8305C) and a normal thyroid follicular epithelial cell line PTFE. GAPDH [file supplementary_figure_2.pdf]

Supplement Figure 4

**A**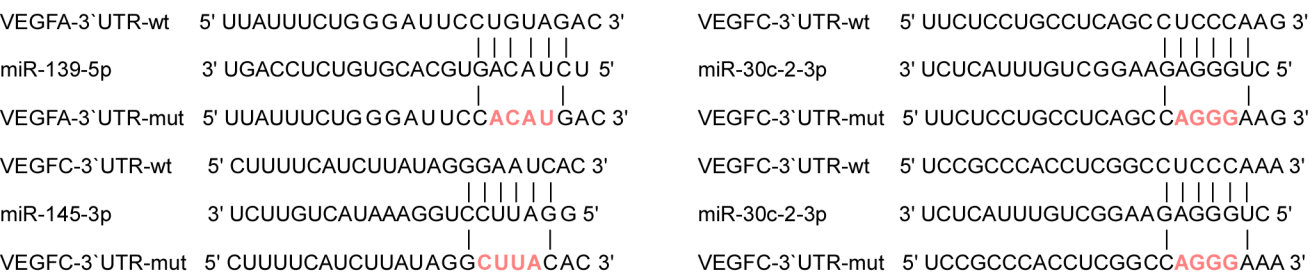**B**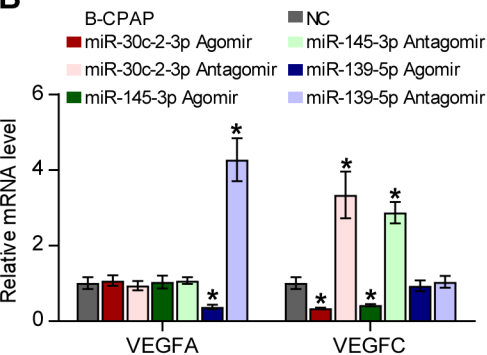**C**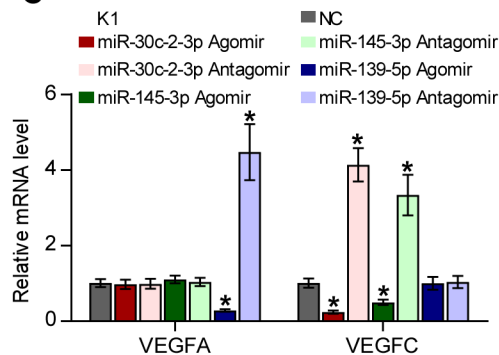**D**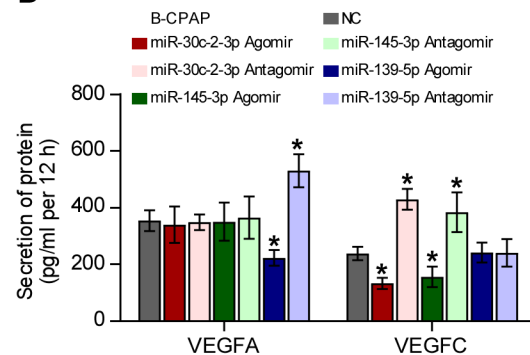**E**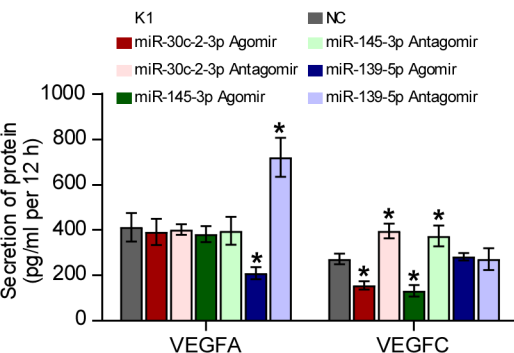**F**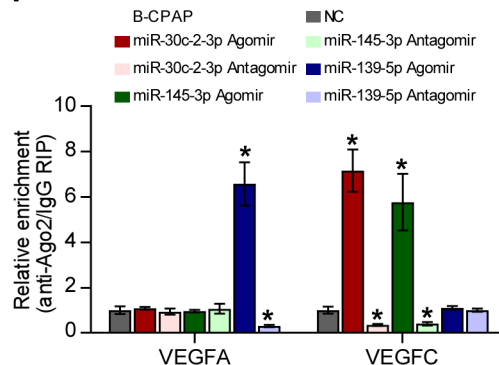**G**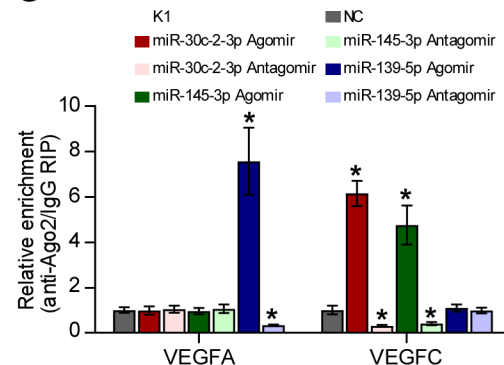**H**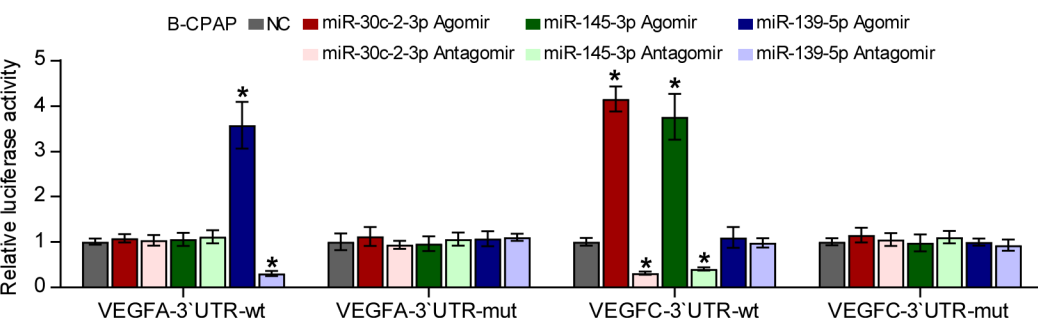**J**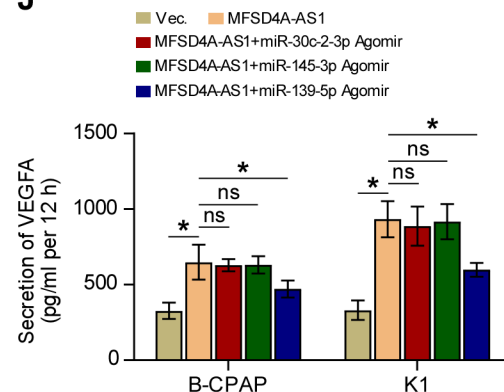**I**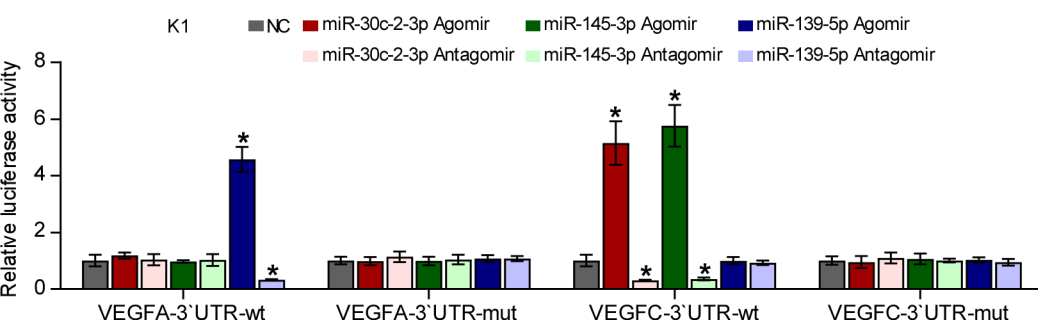**K**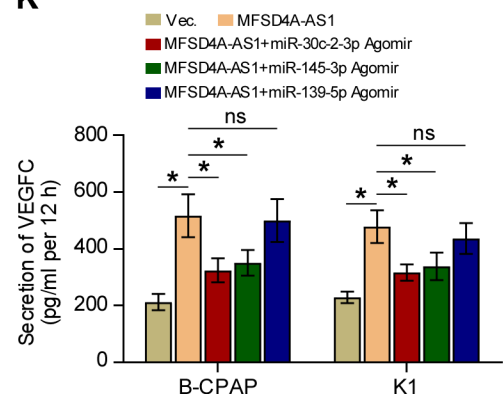

Supplement: Supplemental Figure 4. MFSD4A-AS1 elevates VEGFA and VEGFC dependent on different downstream miRNAs. (A) Predicted miR-139-5p, miR-145-3p, and miR-30c-2-3p targeting sequences and mutant sequences in 3'UTR s of VEGFA and VEGFC. (B and C) Real-time PCR analysis of the effect of miR-139-5p, miR-145-3p [file supplementary_figure_4.pdf]

**Supplement Figure 5****A**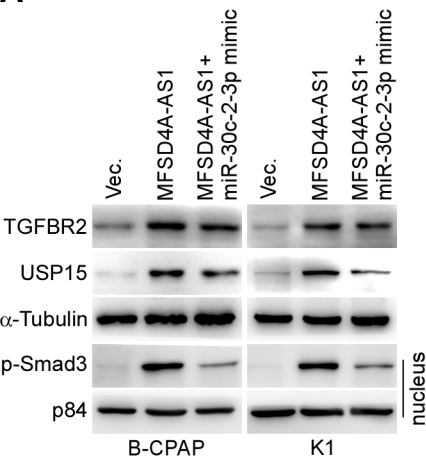**B**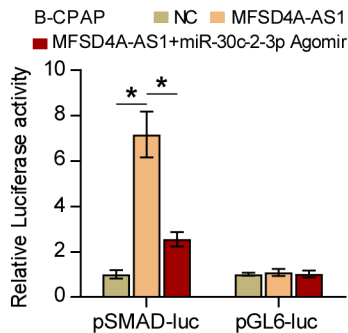**C**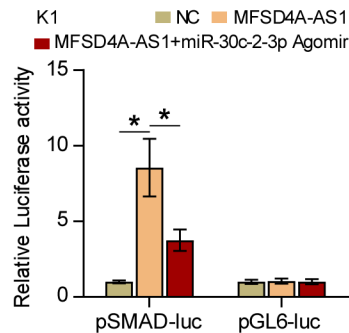**D**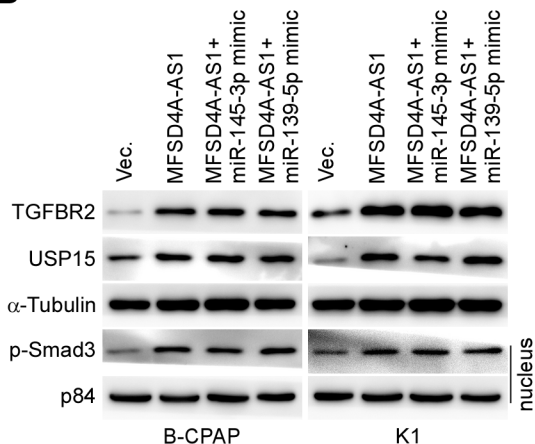**E**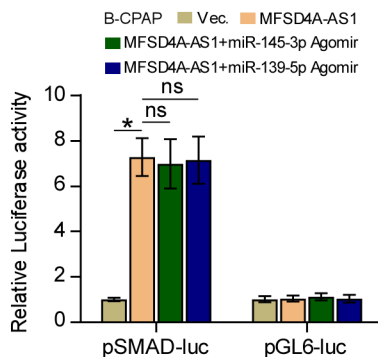**F**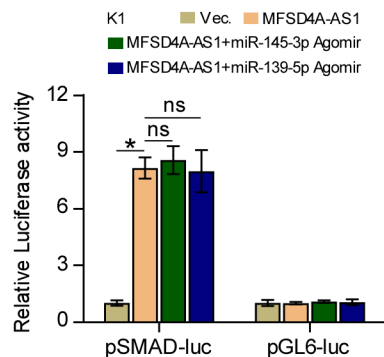

Supplement: Supplemental Figure 5. (A) Western blotting analysis of the effect of miR-30c-2-3p on TGFBR2, USP15 and nuclear translocation of phophorylated Smad3 (p-Smad3) in the indicated PTC cells. α-Tubulin and p84 were served as the cytoplasmic and nuclear loading control respectively. (B and C) The effect o [file supplementary_figure_5.pdf]

# Supplement Figure 6

**A**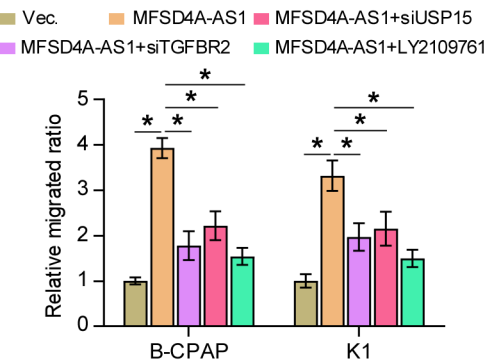**C**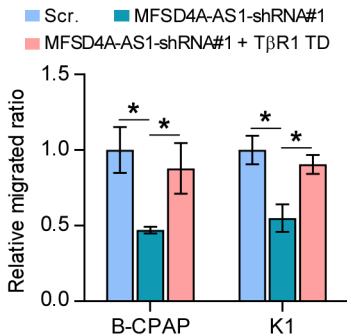**B**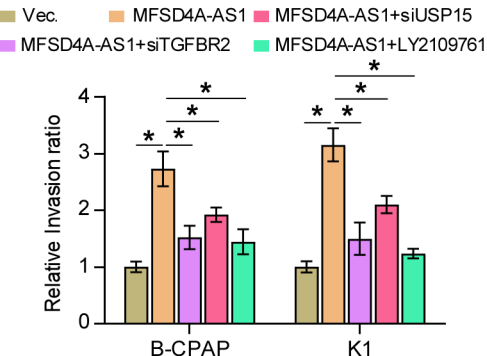**D**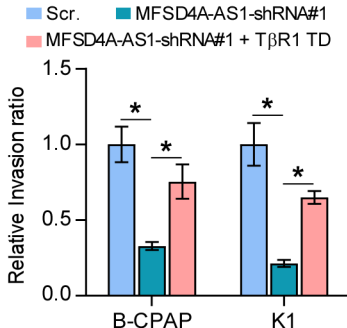

Supplement: Supplemental Figure 6. (A and B) The effect of silencing TGFBR2, USP15 or LY2109761 on migration (A) and invasion (B) abilities of MFSD4A-AS1-overexpressing PTC cells. Each bar represents the mean values ± SD of three independent experiments. *P < 0.05. (C and D) The effect of TβR1 TD, a constitutiv [file supplementary_figure_6.pdf]
