# Supplementary material for: Long non-coding RNA MFSD4A-AS1 promotes lymphangiogenesis and lymphatic metastasis of papillary thyroid cancer
Source: Endocr Relat Cancer. 2023 Feb 8;30(3):e220221. doi: 10.1530/ERC-22-0221 (PMC9986400; doi:10.1530/ERC-22-0221)
Supplement: Supplementary Material [file supplementary_material.pdf]

Supplemental Table 1. The basic information of 102 papillary thyroid carcinoma patients without distant metastasis for MFSD4A-AS1 RNA expression analysis.

		Cases (n)	Percentage (%)
Histologic	PTC	102	100.0
	Other	0	0.0
Gender	Male	10	9.8
	Female	92	90.2
Age	<50	70	68.6
	≥50	32	31.4
T classification	T1	39	38.2
	T2	35	34.3
	T3	22	21.6
	T4	6	5.9
N classification	N0	47	46.1
	N1	55	53.9
M classification	M0	102	100.0
	M1	0	0.0

* PTC: papillary thyroid carcinoma.

Supplemental Table 2. A list of primers used in the reactions for clone PCR.

Gene	Sequence (5` – 3`)
sh MFSD4A-AS1-1#-up	CCGGGCATATCCTGTACCATTTAGGCTCGAGCCTAAATGGTACAGGATATGCTTTTTG
sh MFSD4A-AS1-1#-dn	AATTCAAAAAGCATATCCTGTACCATTTAGGCTCGAGCCTAAATGGTACAGGATATGCT
sh MFSD4A-AS1-2#-up	CCGGGCCTGTATAGCCCACAGAACTCTCGAGAGTTCTGTGGGCTATACAGGCTTTTTG

sh MFSD4A-AS1-2#-dn	AATTCAAAAAGCCTGTATAGCCCACAGAACTCTC GAGAGTTCTGTGGGCTATACAGGCT
MFSD4A-AS1-Luci-up	ATGCAGTGGCATGATGGGTG
MFSD4A-AS1-Luci-dn	TGGGTAGGGACACAGACCTAAAC
VEGFA-3`UTR-up	AGGGTTTCGGGAACCAGATCTC
VEGFA-3`UTR-dn	GCTGGGTTTGTCGGTGTTCC
VEGFC-3`UTR-up	CTGAAGCTTGTCAGCAGTCAAC
VEGFC-3`UTR-dn	CGGTGGCTCACACCTGTAATC

Supplemental Table 3. A list of primers used in the reactions for real-time PCR.

Gene	Sequence (5`– 3`)
MFSD4A-AS1-up	GGCCGAAGCTGAAGAAGACG
MFSD4A-AS1-dn	TTGTGCCTGTGAATAGCCACTG
VEGFA-up	TCTTCCAGGAGTACCCTGATGAG
VEGFA-dn	GCTGGCCTTGGTGAGGTTTG
GAPDH-up	TCCTCTGACTTCAACAGCGACAC
GAPDH-dn	CACCCTGTTGCTGTAGCCAAATTC
VEGFB-up	CAAGTCCGGATGCAGATCCTC
VEGFB-dn	TCTGGCTTCACAGCACTGTC
VEGFC-up	GGCTGGCAACATAACAGAGAA
VEGFC-dn	CCCCACATCTATACACACCTCC
VEGFD-up	TCCCATCGGTCCACTAGGTTTG
VEGFD-dn	ACCACATCGGAACACGTTCAC
PDGFA-up	CGGATACCTCGCCCATGTTC
PDGFA-dn	CTCTCAGGCTGGTGTCCAAAG
FGF2-up	AGTGTGTGCTAACCGTTACCT
FGF2-dn	ACTGCCCAGTTCGTTTCAGTG

EGF-up	TGTCCACGCAATGTGTCTGAA
EGF-dn	CATTATCGGGTGAGGAACAACC
TGFB1-up	CGCGTGCTAATGGTGGAAAC
TGFB1-dn	GCTTCTCGGAGCTCTGATGTG
PDGFB-up	CCCGAGGAGCTTTATGAGATG
PDGFB-dn	GGGAACCCAGGCTCCTTCTT
