# Supplementary material for: Long non-coding RNA MFSD4A-AS1 promotes lymphangiogenesis and lymphatic metastasis of papillary thyroid cancer
Source: Endocr Relat Cancer. 2023 Feb 8;30(3):e220221. doi: 10.1530/ERC-22-0221 (PMC9986400; doi:10.1530/ERC-22-0221)
Supplement: Supplemental Figure 3. (A-C) Predicted recognition sites of miR-139-5p (a), miR-145-3p (b), and miR-30c-2-3p (c) on MFSD4A-AS1. [file supplementary_figure_3.pdf]

Supplement Figure 3

A

```
MFSD4A-AS1-mut   5' ggcacaatCATCATAGUGACAUGc 3'
                            || | | ||||||||
miR-139-5p       3' ugaccucuGU-GCACGUGACAUCu 5'
                            || | | ||||||||
MFSD4A-AS1-wt    5' ggcacaatCATCATACACTGTAGc 3'
```

B

```
MFSD4A-AS1-mut   5' cttACGG-A-TTCGUCCUUACa 3'
                       ||:| | |||||||||||
miR-145-3p       3' ucuUGUCAUAAAGGUCCUUAGg 5'
                       ||:| | |||||||||||
MFSD4A-AS1-wt    5' cttACGG-A-TTCCAGGAATCa 3'
```

C

```
MFSD4A-AS1-mut   5' ccacaATGTACCACAGAGGGUc 3'
                         | ::| |  |||||||
miR-30c-2-3p     3' ucucaUUUGUCGGAAGAGGGUc 5'
                         | ::| |  |||||||
MFSD4A-AS1-wt    5' ccacaATGTACCACTCTCCCAc 3'
```
